# Temperature- and Touch-Sensitive Neurons Couple CNG and TRPV Channel Activities to Control Heat Avoidance in *Caenorhabditis elegans*

**DOI:** 10.1371/journal.pone.0032360

**Published:** 2012-03-20

**Authors:** Shu Liu, Ekkehard Schulze, Ralf Baumeister

**Affiliations:** 1 Laboratory for Bioinformatics and Molecular Genetics, Faculty of Biology, Albert-Ludwigs-University of Freiburg, Freiburg, Germany; 2 Center for Biochemistry and Molecular Cell Research, Faculty of Medicine, and Freiburg Institute for Advanced Studies, School of Life Sciences (FRIAS LIFENET), Albert-Ludwigs-University of Freiburg, Freiburg, Germany; 3 Centre for Biological Signalling Studies (BIOSS), Albert-Ludwigs-University of Freiburg, Freiburg, Germany; Brown University, United States of America

## Abstract

**Background:**

Any organism depends on its ability to sense temperature and avoid noxious heat. The nematode *Caenorhabditis elegans* responds to noxious temperatures exceeding ∼35°C and also senses changes in its environmental temperature in the range between 15 and 25°C. The neural circuits and molecular mechanisms involved in thermotaxis have been successfully studied, whereas details of the thermal avoidance behavior remain elusive. In this work, we investigate neurological and molecular aspects of thermonociception using genetic, cell biological and physiological approaches.

**Methodology/Principal Findings:**

We show here that the thermosensory neurons AFD, in addition to sensing temperature within the range within which the animals can thrive, also contribute to the sensation of noxious temperatures resulting in a reflex-like escape reaction. Distinct sets of interneurons are involved in transmitting thermonociception and thermotaxis, respectively. Loss of AFD is partially compensated by the activity of a pair of multidendritic, polymodal neurons, FLP, whereas laser ablation of both types of neurons abrogated the heat response in the head of the animals almost completely. A third pair of heat sensory neurons, PHC, is situated in the tail. We find that the thermal avoidance response requires the cell autonomous function of cGMP dependent Cyclic Nucleotide-Gated (CNG) channels in AFD, and the heat- and capsaicin-sensitive Transient Receptor Potential Vanilloid (TRPV) channels in the FLP and PHC sensory neurons.

**Conclusions/Significance:**

Our results identify distinct thermal responses mediated by a single neuron, but also show that parallel nociceptor circuits and molecules may be used as back-up strategies to guarantee fast and efficient responses to potentially detrimental stimuli.

## Introduction

Noxious environmental stimuli, such as heat, trigger a survival response in animals resulting in reflexive escape reactions. The first molecular insight into the response to noxious heat came from the cloning and functional characterization of the rodent TRPV1 channel protein, which is activated by capsaicin, the pungent ingredient in hot chili peppers [1]. In mice, TRPV1 is also activated by potentially cell-damaging, noxious temperatures exceeding ∼43°C [1]. However, TRPV1-deficient mice still show a remarkable behavioral response to noxious heat, suggesting the involvement of other heat-responsive proteins in these processes that are TRPV1-independent [2], [3], [4].

The nematode *C. elegans* responds to a wide variety of external stimuli, involving noxious chemicals, high osmolarities, acidic pH, noxious mechanical stimuli, harmful light (UV) and noxious heat [5], [6], [7], [8], [9], but also senses temperature range within which the animals can thrive. *C. elegans* perceives temperatures in the range between 15 and 25°C preferably by using a pair of sensory neurons AFD. These are connected to the AIY interneurons via chemical synapses [10]. This thermotaxis behavior is mediated by a cGMP signaling pathway activating the downstream *tax-2* and *tax-4* CNG channels [11], [12]. In vertebrates, the CNG channels have been implicated in the final step in the G-protein coupled transduction pathways in olfaction and vision [13]. In *C. elegans*, *tax-2* and *tax-4* are required for different sensory sensation, such as the salt sensation in ASE, odorant sensation in AWC, CO_2_ detection in BAG and the temperature sensation in AFD, as well as in the axonal outgrowth of some sensory neurons [14].

The *C. elegans* TRPV channels are also involved in sensory signal transduction. The *osm-9* and five *osm-9*/capsaicin receptor related genes *ocr-1*, *ocr-2*, *ocr-3* and *ocr-4* are coexpressed in sensory cilia and the distinct sensory functions arise from different combinations of *osm-9* and related *ocr* proteins [15]. In AWA and ASH neurons, *osm-9* and *ocr-2* may form heteromeric channels in mediating the chemosensation and nociceptive behaviors, respectively [15].

Our previous experiments established that *C. elegans* displays a pronounced acute withdrawal reaction when subjected to noxious temperatures (∼35–38°C) [8]. Using a local heat source provided by a red diode laser, we defined the head and tail of *C. elegans* as responsive to thermonociception, indicating that the heat-receptive neurons are located in these regions [8]. However, due to the complexity of this nocifensive behavior, the neural circuits involved remain elusive, although from the study of mutants it was concluded that different neural circuits are required for thermotaxis than for thermonociception [8].

We now use laser microsurgery and genetic ablation of cells to identify sensory neurons and circuits in both head and tail that are involved in thermonociception. We find that the AFD thermosensory neurons not only sense temperatures between 15 and 25°C [10], but also contribute to the perception of noxious heat above the threshold temperature 35°C. Elimination of AFD, however, only resulted in a partial defect of the response, since a second pair of sensory neurons in the head, FLP, also function as thermonociceptors. Thermotaxis and thermonociception also differ in the neural circuits to which AFD couple, and use distinct sets of guanylyl cyclases functioning upstream of the CNG channels to mediate specific responses.

Our findings also support a conserved function of the TRPV1 channels OCR-2 and OSM-9 in sensing noxious heat in both mammals and the *C. elegans* FLP and PHC neurons. Both channels act in parallel with the cGMP/CNG pathway in different types of nociceptors. Therefore, noxious temperatures are sensed by neurons in both head and tail, involving circuits which use at least two separate signaling mechanisms. Thus, analogous to the vertebrate nociception, in which TRPV1-dependent and -independent mechanisms are required in different types of C-fibers [16], thermonociception in *C. elegans* is mediated by distinct repertories of ion channels and receptors in different sensory neurons.

## Results

### The head Tav (thermal avoidance) response is mediated by AFD and FLP sensory neurons

**Figure 1:**
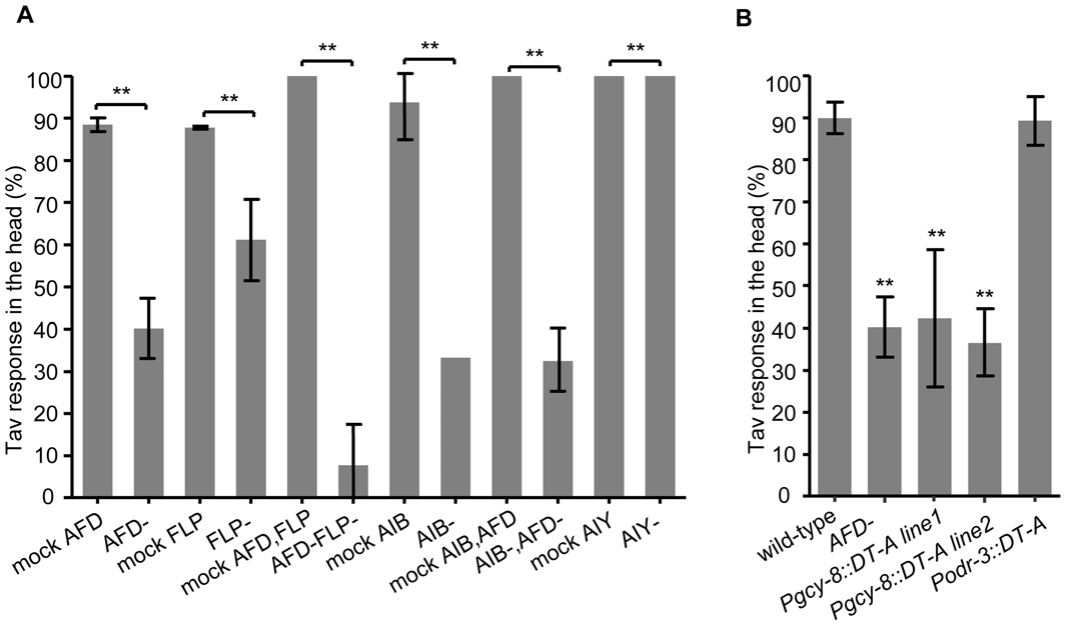
The AFD and FLP sensory neurons mediate Tav response in the head. (A) Laser ablation of AFD, FLP and AIB led to severe defects in the head Tav response compared to mock-ablated animals (n>8). Individual neurons were identified by GFP-labeling and the success of the ablations was visualized by the disappearance of the GFP label in these neurons. Tav responses of the mock-ablated animals slightly differed, which is perhaps due to different GFP transgenes (see Methods). (B) AFD-laser-ablated and two transgenic lines [expressing *Diphtheria* Toxin A (DT-A) under the control of the *gcy-8* promoter] in which AFD was genetically ablated showed defective head Tav response, whereas DT-A killing of five pairs of sensory neurons (from the *odr-3* promoter), but not AFD, behaved like wild-type (n>80). (**P<0.001). Error bars indicate SD.

We directly examined a possible role of the AFD thermosensory neurons in sensing noxious heat by eliminating these and other neurons using microsurgery. For this purpose, we first generated transgenic strains expressing GFP in candidate neurons and ablated individual pairs of GFP-labeled neurons using a 2-photon-laser neuron-ablation approach. We subsequently analyzed the Tav responses of the respective adult animals which had wild-type movements after confirming that the GFP signal in the targeted cells had disappeared following our treatment. Surprisingly, we found a robust decrease of the head Tav responses from 88.5% in mock-ablated animals to 40.2% in animals, in which AFD were ablated (Figure 1A). This suggests that the AFD neurons contribute, in addition to their role in sensing physiological temperatures, also to the sensation of noxious heat. However, the animals' responses were not fully eliminated, suggesting that additional sensory neurons in the head may contribute to the perception of noxious temperature. Based on our previous data that mutants affecting cilia structures showed reduced head Tav responses [8], these other candidate neurons are likely ciliated. Our search focused on the FLP neurons, since a recent report had implicated this pair of multi-dendritic sensory neurons in a variety of nociceptive behaviors [17].

Indeed, FLP ablation reduced the Tav response to 61.1%, and co-ablation of both AFD and FLP together abrogated the head Tav response to 7.8% (Figure 1A and Table S1). Ablation of other ciliated neurons which possess neurites extending into the tips of the nose, like the sensory neurons AWA, AWB, ADF, ADL, BAG, ASH and the PVD nociceptors, which were ablated individually, and the six touch neurons ALMs, PLMs, AVM and PVM which were ablated together, did not affect the Tav response significantly (Table S1). These results indicate that both AFD and FLP contribute to thermonociception, act in parallel and are candidates for heat sensors. Ablation of both pairs of neurons was sufficient to almost entirely eliminate heat response in the head, suggesting that we have identified the two only or, at least, most important pairs of anterior neurons sensing noxious heat.

To confirm these findings using another experimental approach, we also ablated the AFD neurons genetically by expressing the *Diphtheria* toxin A (DT-A) cDNA under the control of the AFD-specific *gcy-8* promoter. Successful ablation was monitored by disappearance of GFP in the AFD neurons (Figure S1). The resulting transgenic strains showed a comparable reduction of the Tav response, whereas expression of the DT-A gene in the AWA, AWB, AWC, ADF and ASH neurons (using the *odr-3* promoter) [18] did not affect the Tav behavior but strongly reduced the nose touch response as an effect of ASH dysfunction (Figure 1B and Table S1, 2). In summary, using two independent methods we show that AFD contributes to mediating perception of noxious heat in the head of *C. elegans*.

The AFD neurons have previously been identified as the major sensory neurons required for the perception of the temperature range within which *C. elegans* can thrive (15°C–25°C), and, together with the AIY interneurons, are part of the thermotaxis neural circuit [19], [20]. Since we found here that AFD is also involved in the sensation of non-physiological, noxious temperatures, it was important to test whether a similar or different neural circuit is involved in the Tav response. For this purpose we laser-ablated AIY and found that the AIY-ablated animals had Tav responses comparable to mock-ablated animals (Figure 1A). Moreover, *ttx-3* mutants, in which the AIY neurons do not differentiate properly [20], also had a head Tav response like wild-type (Table S3). We conclude that neither *ttx-3* nor the AIY neurons are involved in thermonociception, corroborating our previous results that the perception of physiological and noxious temperatures require distinct neural circuits [8].

The other pair of interneurons that connects to the AFD neurons is AIB via electrical junctions [21]. Ablating the AIB resulted in a strongly reduced head Tav response (33.3% vs. 93.8% in mock-ablated animals), which is in the same range of responses of animals in which the AFD neurons were killed (Figure 1A). Ablation of both AFD and AIB together resulted in a Tav response that was not significantly different from the response of the AFD or AIB-ablated animals (Figure 1A, Table S1), indicating that AIB is the major output interneuron of AFD in noxious heat sensation. These results, combined with the fact that AIY and AIB are the only two interneurons connected posterior with AFD [21], led to the assumption that the AFD temperature sensory neurons activate the AIB interneurons upon noxious heat stimulation, whereas they activate the AIY interneurons after sensation of physiological temperatures.

### The Tav response in the tail is mediated by a neural circuit including the PHC sensory neurons and the PVC/DVA interneurons

**Figure 2:**
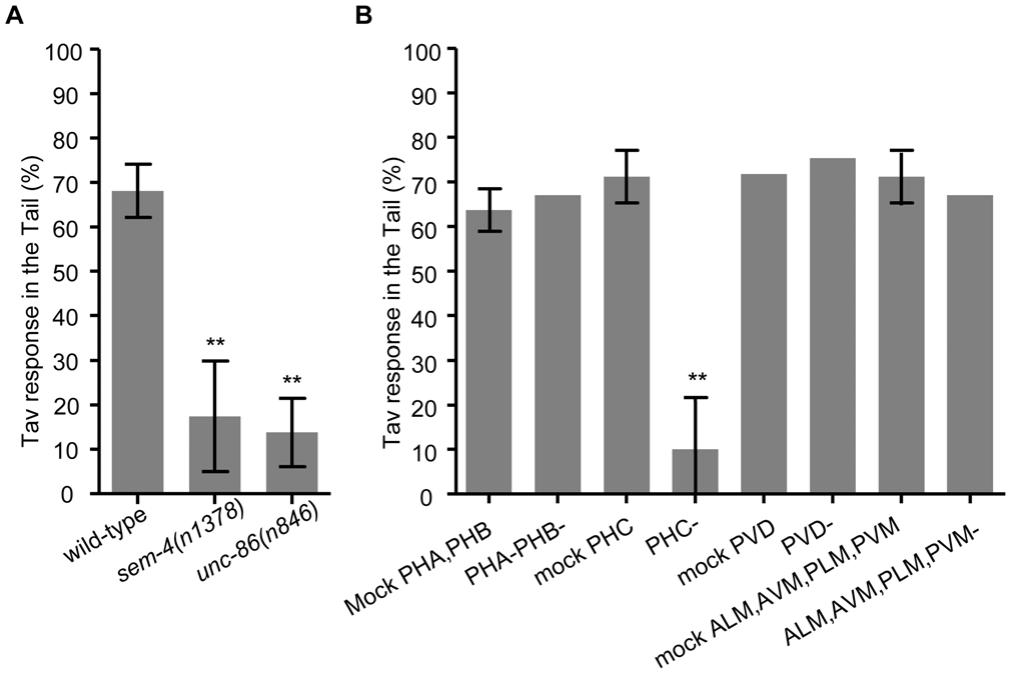
The PHC sensory neurons mediate Tav response in the tail of *C. elegans*. (A) *sem-4* and *unc-86* mutants showed strongly reduced tail Tav response (n>100). (B) Laser-ablation of the PHC neurons almost abrogated the tail Tav response, while ablation of PHA, PHB, PVD and the six touch neurons had no significant defects. (**P<0.001, n>8). Error bars indicate SD.

The avoidance responses of the animals after heat stimulation of the tail are less robust than of the head. Only 68.1% of the animals responded in a reproducible manner to laser stimulation at the posterior end, compared to 95.2% head response. Animals in which both the AFD and the FLP neurons had been ablated showed almost no responses to heat stimuli presented at the head (see above), but responded normally to heat stimuli positioned posteriorly (Table S1). This indicates that heat is sensed by distinct neurons in head and tail, respectively. To identify the latter, we first tested a series of mutants with abnormal development of tail sensory neurons. The POU homeo box gene *unc-86* has a central role in generation of asymmetries within cell lineages and is expressed in one of two daughter cells in 27 lineage classes. We found that the *unc-86(n846)* null mutant showed a severely reduced tail Tav response (Figure 2A and Table S3). A mild reduction of the response to anterior heat stimulation in *unc-86* was reported by us before, which may be caused by the abnormal differentiation of FLP in the mutant, but the tail response had so far not been examined [8]. The development and differentiation of several tail neurons are abnormal in *unc-86* mutant [22], [23]. To further narrow down the list of candidates, we examined *sem-4(n1378)* animals. SEM-4 is a transcription factor acting downstream of UNC-86 in a subset of neurons expressing *unc-86*. Their tail Tav response was reduced to 17.4% that was comparable to that of the *unc-86* mutant. Among the neurons whose development is affected by *sem-4*, only a single pair of sensory neurons, PHC, is positioned posterior to the anal region in the tail of *C. elegans* [24]. Thus, we considered PHC a candidate for a thermonociceptor in the tail. Ablation of PHC was sufficient to reduce the tail Tav response strongly from 71% in mock-ablated animals to 10%, whereas the ablation of other tail neurons, including PVD, PLM, PVM, PHA and PHB, had no significant effect (Figure 2B and Table S2).

Another candidate sensory neuron in the tail is the asymmetric PQR which also expresses *unc-86*. We genetically eliminated PQR, together with the AQR and URX head neurons, using an integrated transgenic line containing *Pgcy-36::egl-1* (a gift from Cori Bargmann, New York) [5]. These animals responded like wild-type to both head and tail heat stimulation, indicating that neither neuron is required for thermonociception. Altogether, these data identify the PHC neurons as a pair of sensory neurons for the perception of noxious temperature in the tail of *C. elegans*. The strong reduction of tail responses after laser ablation suggests that we have identified the major thermonociceptive neurons in the tail.

The main synaptic outputs of PHC are chemical synapses to the PVC and DVA interneurons [24]. To search for the interneurons connecting to PHC in the neural circuit of thermonociception, in a next step we laser-ablated these two interneurons. As reported previously, the tail Tav response is either an accelerated forward movement or a reversal, resulting in a backward escape movement [8]. We found that ablation of either PVC or DVA affected the Tav response profoundly, since 8/19 (DVA) and 12/18 (PVC) animals did not respond at all to heat applied to the tail. However, none of the PVC-ablated animals but 11 of the DVA-ablated animals had maintained their ability to accelerate, and none of the DVA-ablated animals but 6 of the PVC ablated animals showed backing responses (Table 1). These data are corroborated by the behavior of the *deg-1(u38)* mutant, in which the PVC neurons degenerate [25]. The occasional responses of these animals to heat at the tail were exclusively backward, and no forward movement was observed (Table S3). These results suggest that, in addition to PHC that are thermonociceptive sensory neurons, the interneurons PVC and DVA are part of the neural circuit responding to heat at the tail. PVC mediates accelerated forward response in forward moving animals, whereas DVA triggers a reversal of resting animals.

**Table 1:** The PVC and DVA interneurons mediate thermonociception in the tail of *C. elegans*.

Neurons ablated	No. of animals showing forward tail Tav response	No. of animals showing backward tail Tav response	No. of animals tested
mock-ablated (DVA)	6	3	10
DVA-ablated animals	11	0	19
mock-ablated (PVC)	6	6	13
PVC-ablated animals	0	6	18

### AFD, FLP and PHC are activated by noxious heat

Our results obtained so far only suggest, but do not prove that AFD, FLP and PHC respond to heat as sensory neurons. To measure heat-induced activity in these neurons directly, we imaged neuronal calcium influx *in vivo* using the calcium indicator protein cameleon, which emits increased fluorescence ratio of YFP to CFP upon calcium binding [26].

Previous results have suggested that a transient increase in the YFP/CFP fluorescence ratio in cameleon YC2.12 in the AFD neurons can be observed upon warming from 15°C to 25°C at a 1.5°C/s ramp rate, indicating the response of AFD to a temperature change in the physiological range during thermotaxis [10]. Here, to simulate the noxious heat stimulus used in the Tav assay, we used an experimental setup allowing a very fast temperature change from ∼22°C to ∼38°C with a ramp rate of 8°C/s. With the steep temperature gradient, the animals show rather the avoidance response than the thermotaxis behavior [27].

We expressed cameleon in different sets of neurons. The Tav responses of these transgenic animals were analyzed since calmodulin as a calcium sensor participates in a variety of intracellular processes after conformational change upon binding to calcium [26]. For all the transgenic animals, wild-type head Tav responses and slightly reduced tail Tav responses were observed (Table S4); indicating that expression of cameleon has no effect on the heat perception in the head and impairs the tail Tav response only marginally, which may be the consequence of cameleon protein expression.

**Figure 3:**
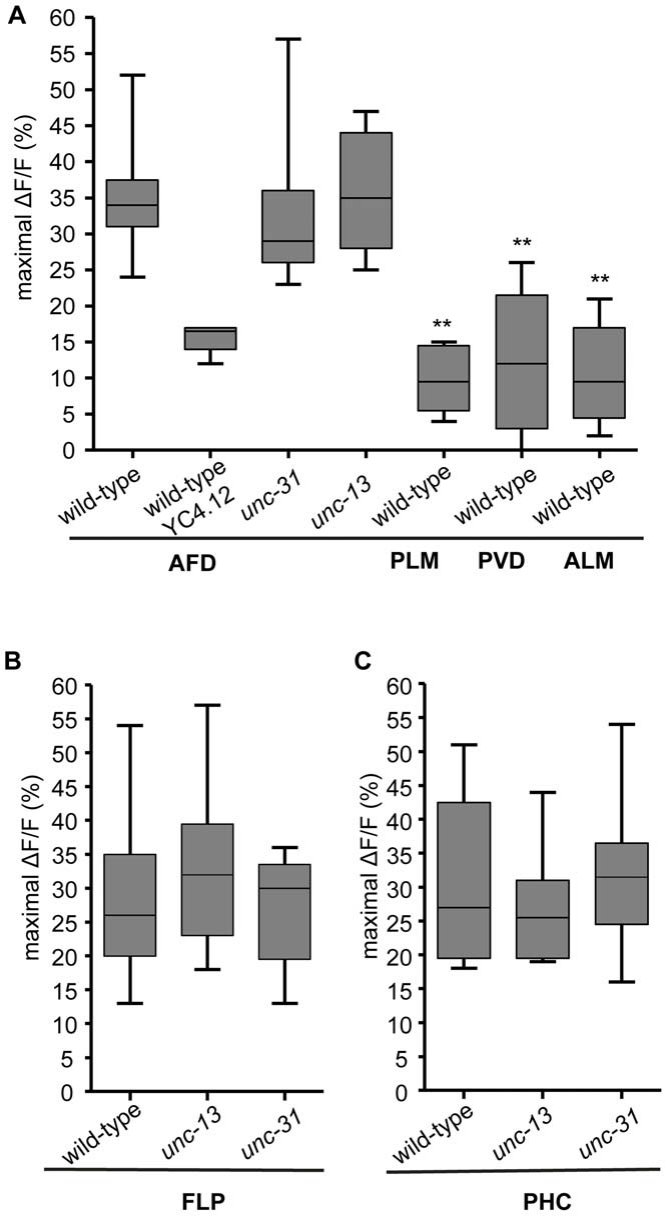
AFD, FLP, and PHC function as primary sensory neurons in the Tav response. (A) Average ratio changes in AFD, PLM, PVD and ALM upon noxious heat. (**P<0.001 different from ratio changes in AFD in wild-type). (B), (C) Average ratio changes in FLP and PHC upon heat stimuli in wild-type, *unc-13* and *unc-31* mutant backgrounds. Data are shown in box plot (n>4). Error bars indicate SD.

We used a Nikon A1 confocal microscope to record the FRET signal. Compared to the sensitized CCD system used in previously published experiments [28], we noticed that the photomultiplier-based signal recording had a lower signal-to-noise ratio. In Figure S2, four representative traces of AFD, FLP and PHC neuron responses to the noxious heat stimulus are shown. Large calcium transients with a 35.8% ratio change were observed by heat stimulation in the AFD cell bodies, compared to 10.4%, 9.7% and 12.8%, respectively, measured in the ALM, PLM and PVD neurons (Figure 3A and Table 2). Therefore, rapid temperature changes to 38°C typical for thermal avoidance response induce calcium signaling in AFD, but not in ALM, PLM and PVD. YC4.12 is another cameleon protein with a lower affinity for calcium [10]. To exclude that the conformational change of cameleon itself resulted in altered YFP/CFP ratio upon heat, but not calcium binding, we also tested animals expressing YC4.12 in AFD. The resulting ratio change was less than half of that observed with YC2.12 (Table 2), suggesting that the FRET signal we observed was indeed due to changes of the intracellular calcium concentration in AFD. We also measured calcium signaling in the FLP and PHC neurons using the same protocol as in AFD. Robust transient calcium increases were observed in both FLP (29.3%) and PHC (30.5%; Figure 3B, C and Table 2) that correspond to those recently reported for the FLP sensory neurons using a different protocol [29]. Together, our data indicate that the AFD, FLP and PHC sensory neurons are activated by rapid temperature shifts from 22°C to 38°C, the latter being considered noxious for *C. elegans*.

**Table 2:** YFP/CFP ratio change (calcium influx) after noxious heat stimuli in different sensory neurons.

Genotype	AFD(yc2.12)	FLP(yc2.12)	PHC(yc2.12)	AFD (yc4.12)	ALM(yc2.12)	PLM(yc2.12)	PVD(yc2.12)
N2 (wild-type)	35.8±9.6 (26)	29.3±13.0 (12)	30.5±11.6 (14)	15.3±2.5 (4)	10.4±6.8 (10)	9.7±4.3 (12)	12.8±9.4 (9)
*unc-13(e450)*	35.8±8.4 (10)	32.9±12.0 (13)	26.3±7.8 (10)	ND	ND	ND	ND
*unc-31(e928)*	31.4±8.5 (17)	26.8±7.7 (13)	32.2±10.1 (12)	ND	ND	ND	ND

Five seconds after recording, the animals were exposed to the noxious heat stimuli which reached 38°C maximal temperature after seven seconds. Values reported are mean % ± SD % (sample size) of the average maximal FRET ratio change amplitude in each population post stimuli. ND: not determined.

Next, we gathered further evidence to support the hypothesis that AFD, FLP and PHC act as sensory rather than accessory neurons in the neural circuit of thermonociception. We reasoned that blocking neurotransmission should not affect calcium responses in primary sensory neurons, but should reduce or block heat-evoked calcium transients in these neurons, if they serve as accessory neurons and the activation of which is dependent on neurotransmission. The gene *unc-13* regulates exocytosis of synaptic vesicles, and, thus, neurotransmitter release, whereas *unc-31* encodes the *C. elegans* CAPS homolog necessary for the exocytosis of dense core vesicles [30], [31]. Heat-evoked calcium transients in both mutants are not significantly decreased in either the AFD, FLP or PHC neurons (Figure 3A, B, C and Table 2). Therefore, we suggest that the AFD, FLP and PHC do not depend on synaptic and dense core vesicle neuronal transmission for activation by noxious heat. Considering these conclusions and the fact that these sensory neurons have only synaptic connections but no gap junctions which mediate electrical transmission with other sensory neurons [21], we suggest that AFD, FLP and PHC are primary sensory neurons responding directly to noxious temperatures.

### TRPV1 channels contribute to the Tav response in the FLP and PHC sensory neurons

**Figure 4:**
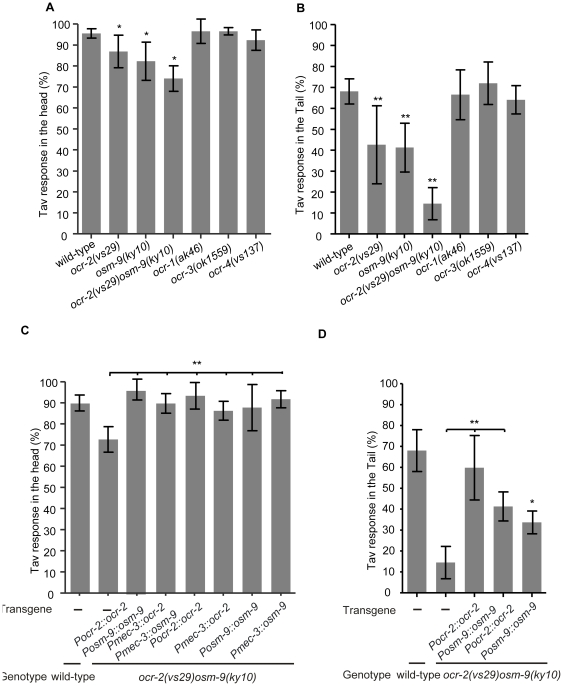
OCR-2 and OSM-9 contribute to the Tav response in the FLP neurons in the head and in the PHC neurons in the tail of *C. elegans*. (A), (B) The head and tail Tav of *osm-9* and *ocr* single and double mutants are shown. (C) The defective Tav response in the head of the *ocr-2 osm-9* double mutant was rescued by the expression of *ocr-2* and/or *osm-9* full length genomic DNA as well as by expression of their respective cDNAs under the control of the *mec-3* promoter. (D) The expression of either *ocr-2* or *osm-9* full-length genomic DNA was sufficient for at least partial rescue of the defective Tav response in the tail of the *ocr-2 osm-9* double mutant. (*P<0.01; **P<0.001, n>80). Error bars indicate SD.

The human TRPV1 channel is known to be directly activated by noxious stimuli, such as heat or acidic pH [1]. However, in vertebrates the TRPV1 channel is typically activated at temperatures above 43°C. We asked whether the function of TRPV1 in sensing noxious temperatures is conserved in *C. elegans*. Using sequence analysis software based on a Hidden Markov model, we found ten homologues of TRPV channel genes in *C. elegans* (Figure S3), including the OSM-9 and the four OCR proteins studied previously [15]. Of these, *unc-44* encodes a family member that only contains ankyrin repeats, but no ion channel domain. As a consequence, we excluded *unc-44* but investigated the Tav response of mutants in all other TRPV channels. Mutants of *ocr-2* and *osm-9* had mildly reduced Tav response, while the other three *ocr* mutants (*ocr-1*, *ocr-3*, *ocr-4*) (Figure 4A) and *trp* mutants (*trp-1*, *trp-2*, *trp-4*, *trpa-1*) had Tav response like wild-type (Table S5). The *ocr-2(vs29) osm-9(ky10)* double mutant showed a more severe reduction in the head Tav response (Figure 4A), and displayed a drastically reduced tail Tav response (Figure 4B and Table S5). We conclude that both *ocr-2* and *osm-9* contribute to the behavior. These data are consistent with TRPV channels sharing a common function in the response to noxious temperatures in both *C. elegans* and mammals.

Both the *ocr-2* and *osm-9* genes are expressed in PHC and at least *osm-9* is expressed in the FLP sensory neurons but not in AFD [15], [32]. We next asked whether expression of *ocr-2* and *osm-9* in FLP and PHC is sufficient for rescuing the defective Tav response in the mutant. For this purpose we generated genetic mosaic animals expressing *ocr-2* and *osm-9* cDNAs in subsets of cells of the *ocr-2(vs29) osm-9(ky10)* double null mutant. Since no promoters driving expression specifically in the FLP and PHC neurons were available, we used the *mec-3* promoter for FLP expression and the respective endogenous promoters for the expression in PHC. The *mec-3* promoter is active in FLP, PVD, and the six touch receptor neurons [33]. As described above, animals in which the six touch receptor neurons or the PVD neurons were ablated showed wild-type Tav response (Table S1), suggesting that these neurons do not play roles in thermonociception. Expression of either *ocr-2* or *osm-9*, or both together, under the control of the *mec-3* promoter (in extrachromosomal arrays) was sufficient to yield full rescue of the Tav response defect. The level of rescue was comparable to that obtained with genomic copies of *ocr-2* and/or *osm-9* genomic DNA. We conclude that expression of either of both TRPV channel genes is sufficient for the FLP neurons to respond to heat. This result provides additional support for redundant functions of *ocr-2* and *osm-9* (Figure 4C and Table S5). It is noteworthy that, although a *Pocr-2::gfp* expression was not observed in FLP [15], [32], our genetic data support a function of *ocr-2* in these neurons.

In the tail of *C. elegans*, *ocr-2* and *osm-9* expression only overlaps in the PHA, PHB and PHC neurons [32]. To address whether OCR-2 and OSM-9 have similar function in the posterior PHC neurons, transgenic lines expressing *ocr-2* or/and *osm-9* genomic DNA under the control of the endogenous promoters in an *ocr-2 osm-9* double mutant background were analyzed. Significant increase in the tail Tav response was observed when both genes were expressed, whereas expression of either *ocr-2* or *osm-9* genomic DNA led to partial but significant rescue of the defective tail Tav response in the double mutant (Figure 4D). Since we have shown that PHA and PHB do not participate in the tail Tav response, our data further support the role of *ocr-2* and *osm-9* in the PHC neurons. In summary, our data suggest that *ocr-2* and *osm-9* both mediate Tav responses in two pairs of sensory neurons, FLP in the head, and PHC in the tail. There is no evidence that both genes are involved in thermonociceptive responses mediated by AFD.

### The head Tav response is mediated by a cGMP signaling pathway in the AFD neurons

**Figure 5:**
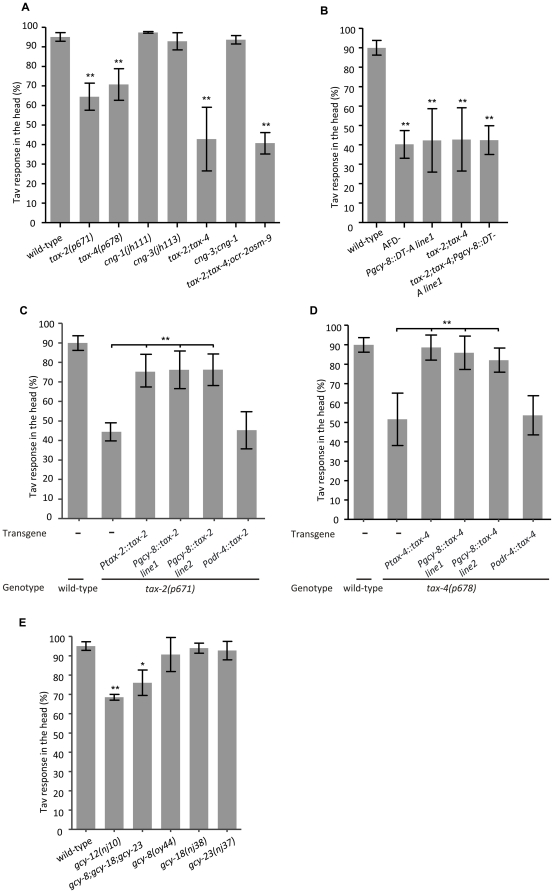
A cGMP signaling contributes to the Tav response in the AFD neurons. (A) The head Tav responses of mutants in CNG channel genes are shown. (B) Genetic-ablation of AFD did not further decrease the head Tav response in *tax-2*;*tax-4* double mutant. (C), (D) The defective Tav phenotype of both *tax-2* and *tax-4* single mutant was rescued by expressing a wild-type copy of the respective gene (*tax-2* or *tax-4*). Expression of *tax-4* or *tax-2* cDNA under the control of the AFD-specific *gcy-8* promoter rescued the respective mutant phenotype. No rescue was obtained when the *tax-4* or *tax-2* cDNA was expressed from the *odr-4* promoter (expressed in 12 neurons, but not in AFD). (E) The head Tav responses of the *gcy* mutants are shown. (*P<0.01; **P<0.001, n>50). Error bars indicate SD.

In the search for candidate proteins sensing heat or transmitting heat-induced stimuli in the AFD neurons, we focused on the CNG proteins that are expressed in several sensory neurons including AFD but not FLP [11], [34], [35]. There are six CNG channel protein homologues encoded in the *C. elegans* genome, mutants are available for four of them. We tested both *cng-1(jh111)* and *cng-3(jh113)* single mutant alleles that are predicted as null alleles, together with the double mutants. They all showed responses similar to wild-type (Figure 5A), indicating that these genes are not involved in the Tav response. The CNG channel subunits *tax-2* and *tax-4* are implicated in mediating thermotaxis in AFD [11]. We had previously reported that the *tax-2(p694)* weak mutant allele [36] showed wild-type Tav response and that *tax-4(p678)* null mutant [11] only resulted in a slightly reduced Tav phenotype [8]. A careful reexamination of the *tax-4(p678)* mutant after four additional backcrossings to wild-type revealed a more reduced head Tav response than previously reported (Figure 5A). Additionally, two loss of function mutant alleles *tax-4(ks28)* and *tax-4(ks11)* were analyzed and defective head Tav responses similar to *tax-4(p678)* were observed in these mutants (Table S6). We also reexamined the role of *tax-2* by testing the other three loss of function alleles *tax-2(p671)*, *tax-2(ks10)* and *tax-2(ks31)* [36] that we had not analyzed before. These alleles showed strongly reduced response (Figure 5A and Table S6). Part of the discrepancy to our earlier results may have arisen from the fact that we previously only reported those worms as defective that have completely lost any response to noxious heat [8]. In the meantime we realized that certain mutants, although eventually responding to prolonged exposure to temperatures above 35°C, displayed defects in certain aspects of the complex Tav behavior, and/or showed for example prolonged retardation time periods. Although there is no evidence that *tax-2* and *tax-4* are expressed in any tail neurons, mutations in both genes also significantly reduced the tail Tav response (Table S6), suggesting the possible function of these channel proteins in the posterior part in sensing noxious heat. Moreover, the double mutant *tax-2;tax-4* further reduced the Tav response in the head to 42.8% and in the tail to 36% (Figure 5A and Table S6). The role of *tax-2* and *tax-4* in thermonociception is probably limited to AFD, since genetic ablation of AFD in a *tax-2;tax-4* mutant background did not further increase the Tav defect (Figure 5B). Although mutants in the additional two genes of the CNG family were not available, their contribution to AFD function is less likely, given that *tax-2;tax-4* already have defects as strong as those observed in AFD ablated animals (Figure 5B).

Defects of both *tax-2* and *tax-4* were rescued by reintroducing a wild-type copy of each gene into the respective mutant (Figure 5C, D and Table S6). The AFD-specific role was addressed by expressing *tax-4* or *tax-2* cDNAs under the control of the AFD neuronal specific *gcy-8* promoter which proved to be sufficient for rescuing the mutant defects (Figure 5C, D). In contrast, expression of the *tax-4* or *tax-2* cDNA from the *odr-4* promoter which is active in many other ciliated neurons (AWA, AWB, AWC, ADF, ASG, ASH, ASI, ASJ, ASK, ADL, PHA, PHB), but not in the AFD neurons [37], did not rescue the phenotype of the corresponding mutant (Figure 5C, D and Table S6). These data support our claim that *tax-2* and *tax-4* are required cell-autonomously in the AFD sensory neurons to mediate thermonociception in the head of *C. elegans*.

It has been shown that the four guanylyl cyclases *gcy-8*, *gcy-18*, *gcy-23* and *gcy-12* are expressed in the AFD neurons and *gcy-8*, *gcy-18*, *gcy-23* are required upstream of the CNG channels in thermotaxis [38]. To elucidate whether these four guanylyl cyclases also contribute to the signal transduction of thermonociceptive stimuli in AFD, we tested these mutants. Only the *gcy-12* single mutant showed a strongly reduced head Tav response, whereas the *gcy-8*, *gcy-18* and *gcy-23* single mutants showed wild-type behavior. However, it is worth noting that the *gcy-8 gcy-18 gcy-23* triple mutant did display a mildly reduced Tav response (Figure 5E). These results imply a signaling role of the guanylyl cyclases GCY-12, and, to a lesser extent, of GCY-8, GCY-18 and GCY-23 upstream of the CNG channels TAX-2 and TAX-4. This signaling differs in several aspects from that observed in the sensation of physiological temperatures, since a different ranking of individual guanylyl cyclases is observed, and distinct interneurons, and, thus, selective different neural circuit and signaling pathway are used to mediate the Tav response.

We conclude that, in addition to the OCR-2 and OSM-9 TRPV channels with functions in FLP and PHC, the CNG channels TAX-2 and TAX-4 mediate Tav response in AFD, and have a previously not documented role in noxious heat perception in the posterior part of *C. elegans*. TAX-2 and TAX-4 activity in the tail may require OCR-2 and OSM-9 function, since *ocr-2 osm-9* double mutant, with a stronger defect in the tail Tav response than *tax-2;tax-4*, is not further enhanced in the *tax-2;tax-4;ocr-2 osm-9* quadruple mutant (Table S6). Our data also suggest that there are other proteins involved in the sensation of the FLP-specific heat response, since the quadruple mutant *tax-2;tax-4;ocr-2 osm-9* only reduced the head Tav response to a level observed in *tax-2;tax-4* alone, but was weaker than the laser ablation of both AFD and FLP (Table S6).

## Discussion

**Figure 6:**
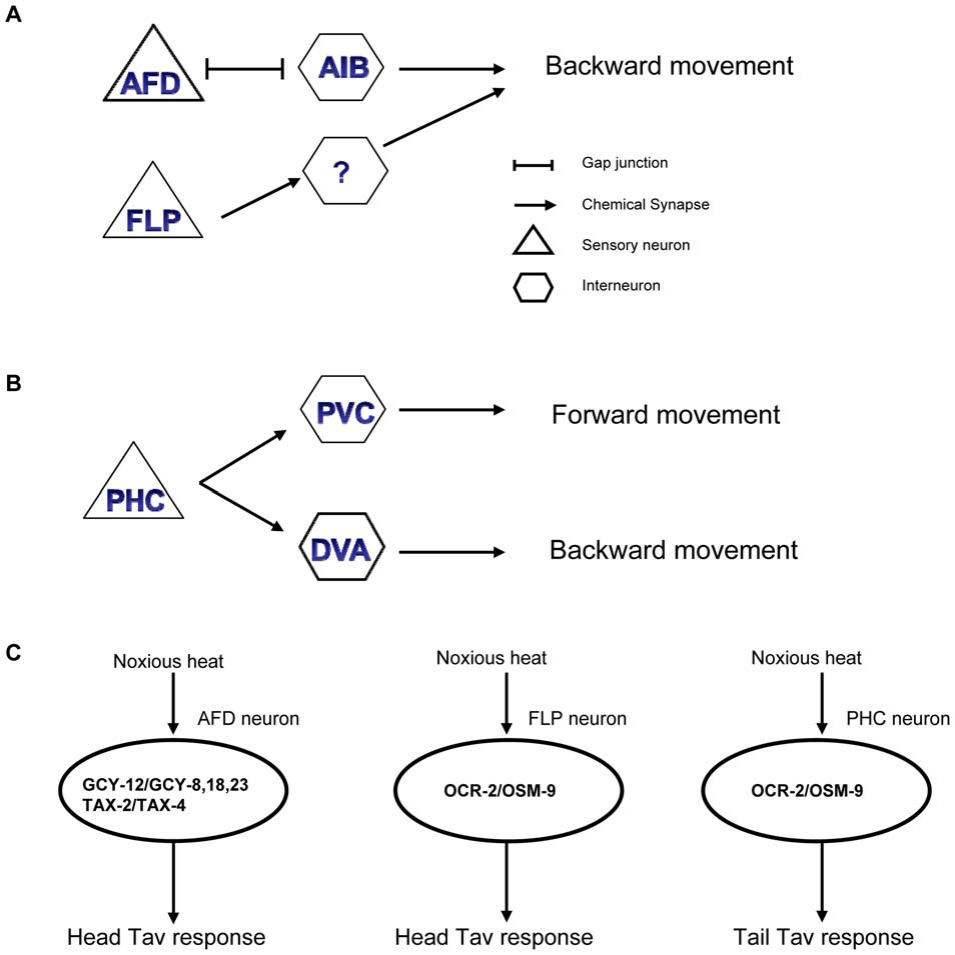
Models of noxious heat sensory neuron function. (A) Circuit diagram of head Tav response. AFD forms gap junction with AIB. (B) Circuit diagram of tail Tav response. (C) Genetic pathways contributing to Tav response. Head Tav response is mediated by a cGMP signaling pathway within the AFD neurons and a TRPV1 pathway in FLP, whereas the tail Tav response is mediated via TRPV1 in the PHC neurons.

We identified here the FLP and AFD neurons in the head and the PHC neurons in the tail of *C. elegans* as thermonociceptive sensory neurons. The AFD neurons have a complex anatomy with numerous finger-like cilia [39], a structure to enlarge its surface and to facilitate temperature sensation. The AFD neurons are polymodal, responding in addition also to temperature alterations in the physiological range, and act as CO_2_ sensors [40]. The second pair of thermonociceptor neurons, the FLP neurons, are also polymodal and respond in addition to noxious mechanical stimuli [6]. Their neurites develop into a complex branched network of thin sensory processes, similar to the PVD nociceptors [17]. Branched, tree-like cilia are characteristics of certain types of nociceptors in vertebrates [16], and also *Drosophila* polymodal nociceptors are attached to epidermal cells with multiple branched naked nerve endings [41]. In our thermal avoidance assays, only the FLP, but not the PVD neurons of *C. elegans* responded to direct exposure by a local source of heat. The PVD neurons, in contrast, have been shown before to get stimulated by acute cold shock [29]. AFD receives synaptic inputs from AIN and AWA and has gap junctions to AIB and itself. FLP receives synaptic input from ADE and connects with RIH, AVD and itself via gap junctions [21]. Based on the fact that the activation of AFD and FLP upon noxious heat does not depend on neuronal transmission (that can be blocked by *unc-13* and *unc-31*), the synaptic connections between AIN, AWA and AFD, ADE and FLP are probably not involved in heat sensation. Besides AFD and FLP, there were no further sensory neurons identified as head thermonociceptors in this study. Moreover, ablation of both pairs of neurons almost abrogates the head Tav response. Altogether, we assume that AFD and FLP are the primary anterior neurons sensing noxious heat (Figure 6A). Although, from the data shown here, the existence of a more complicated neural circuit, in which AFD and FLP serve as accessory neurons, accepting the neuronal information via gap junctions, could not be fully excluded. A third pair of thermonociceptive neurons, PHC, is localized in the tail of the animals, and we suggest that PHC, together with the two asymmetric interneurons DVA and PVC, executes the complex tail response to heat. Animals in forward motion accelerate their speed to avoid a local heat source placed posteriorly, whereas resting animals typically move backwards [8]. Ablation of PHC almost entirely eliminated the heat responses in the tail, suggesting that PHC is the major posterior thermonociceptive neuron. The involvement of DAV and PVC interneurons is more complex. Both neurons have been implicated previously as interneurons in tail touch responses, PVC as a tail touch modulator, and DVA as a mediator of responses to harsh touch (Tap reflex) [42]. Ablation of each neuron prevented any response in a considerable portion of animals, making a functional assessment difficult. However, since no animal in which DVA was ablated showed backward movement, and no animal in which PVC was eliminated displayed accelerated forward movement upon tail stimulation, our data are consistent with these two neurons playing opposing roles (Figure 6B). Based on the wiring diagram, PVC has synaptic connections with AVD, AVB and AVE interneurons and gap junction with AVJ. DVA has synaptic connections with AVA, AVE and gap junctions with AVB [21]. We observed that the Tav withdrawal is similar in its behavioral consequences to the Tap reversal reaction, resulting in a 180°C inversion of the movement direction. Since during the Tap response, PVC and AVB are required for forward movement after tail touch, whereas AVD and AVA are required for backward movement in the head touch subcircuit [42], we speculate that for the Tav response, PVC may trigger forward movement by activating AVB, whereas DVA may trigger reversals by chemical synapsing to AVA. Further neuron ablation and calcium imaging experiments may help to understand the neural circuits in more detail.

Our data strongly imply that the AFD neurons are thermosensors responding to distinct ranges of temperature. While small temperature changes that deviate from cultivation conditions transiently activate AFD and provoke a response involving the AIY interneurons to which they directly couple [10], we find that the Tav response involves the AIB interneurons, suggesting a distinct neural circuit of thermoavoidance. Unlike thermotaxis, the response to noxious heat requires a fast neurotransmission in order to prevent tissue damage, and, this may be the reason why the AFD neurons couple to AIB via gap junctions. An obvious function of the gap junctions is to mediate electronic transmission which allows propagating rapid impulses. Especially in cold-blooded animals, the electronic transmission is significantly faster than chemical transmission, and is found in the escape mechanisms in Aplysia [43]. We therefore speculate that gap junctions might play a more prominent role in the thermoavoidance than in the thermotaxis response of *C. elegans*.

Our genetic data strongly implicate that at least two channel protein families contributed to thermonociception in *C. elegans* in distinct neurons: the TRPV and the CNG channels. The vertebrate TRPV1 channel is activated by noxious heat, acids, and capsaicin [1]. A conserved function in noxious heat sensation is shared by two of the *C. elegans* TRPV channels, OSM-9 and OCR-2. The involvement of TRPV channels in thermonociception also supports the notion that wild-type animals show hyperalgetic Tav reaction after capsaicin treatment [8]. OSM-9 and OCR-2 have been characterized to assemble into a heteromultimeric channel complex in ASH and AWA, where they function in signal transduction in the neuron-mediated nociceptive and chemotaxis behaviors [15]. We suggest here a similar model for the sensation of noxious heat in FLP. However, although the defective Tav response in *ocr-2 osm-9* is significant, the phenotype observed in FLP-ablated animals is more severe (Table S3, 4), suggesting the involvement of other, so far undetected factors besides OCR-2 and OSM-9 in thermonociceptive signal transduction in the FLP neurons.

Recently, an independent study reported by Glauser and colleagues also identified a minor contribution of single and double combinations of *C. elegans* TRPV channel in crossing noxious temperature barriers, corroborating our findings [44]. These experiments capitalized on the attraction of *C. elegans* to certain odorants, which actively forces the animals to overcome a local adversive temperature barrier or gradient. In these assays, ablation of the AFD neurons did not affect the response of *C. elegans*, suggesting that the response from this assay is distinct from our Tav response, or the interference between heat avoidance and chemical attraction may be more difficult to dissect than anticipated. In addition, these assays require starving the animals six hours prior to analysis, which is not a typical prerequisite for a nociceptive response and may actually modulate heat avoidance behavior. In fact, our own previous results indicate that animals that have been starved in order to enter the dauer stage had lost their Tav response almost entirely [8], and genetic modulation of the insulin signaling pathway that is affected by starvation showed altered Tav responses (our unpublished observations). Moreover, the laser ablation of ASH and AWA chemosensory neurons even slightly increased the head Tav response compared to the mock-ablated animals (Table S1), which may indicate a negative modulation of thermonociception through activation of chemosensory neurons. Finally, using our assays we are able to show that Tav responses differ in head and tail, which are possible to be dissected by using a local heat source rather than a gradient. It is currently not known how *C. elegans* integrates noxious sensory inputs from both head and tail. In comparison, the tail sensory neurons PHA and PHB antagonize the ASH and ASK head sensory neurons to affect SDS chemical avoidance response [45]. We therefore suggest that the combination of Tav assays described here and thermal barrier and thermogradient assays described elsewhere [44] should be useful in understanding different aspects of heat avoidance responses.

In addition to the TRPV channels, *C. elegans* obviously uses also the two CNG channels TAX-2 and TAX-4 that function cell-autonomously in the sensation of noxious heat in the AFD neurons. *tax-2* and *tax-4* encode the β- and α-subunits of a CNG channel complex; α-subunits could either form channels on their own or form heteromultimeric channels with β-subunits [36]. The stronger defect in *tax-2;tax-4* double mutant compared with single mutants (Figure 5A) suggests that TAX-4 may function both alone and also in a complex with TAX-2 in AFD to mediate Tav response. TAX-2 and TAX-4 have been shown to be required in olfaction, thermosensation, and axon outgrowth [7]. We do not consider a developmental defect of TAX-2/TAX-4 to be the cause of reduced Tav response, since no defect in the AFD morphology was observed in the *tax-2* and *tax-4* mutants [36]. Our genetic data suggest that upon noxious heat, TAX-2 and TAX-4 were activated by cGMP mainly generated in the AFD neurons through the activity of *gcy-12*. Although GCY-12 activity has been shown to be clearly dependent on temperature [38], it is non-relevant for the thermotaxis behavior [38]. Thus, albeit the same sensory neurons (AFD) and CNG channel proteins (TAX-2, TAX-4) contribute to thermotaxis [11] and thermal avoidance, selective differences are employed in the sensation and response to physiological and non-physiological temperatures at the neuronal and molecular level. *gcy-8, gcy-18* and *gcy-23* are mainly required in sensing ambient temperature, whereas *gcy-12* is only involved in sensing noxious heat. Recently, it has been shown that the G protein-coupled receptor (GPCRs) STRX-1 is required both in AFD and AWC for retaining normal isothermal tracking behavior (Ikue Mori, personal communication). We tested the *strx-1* mutant and detected no defect in the Tav response, indicating the involvement of *strx-1* specifically in sensing ambient but not in noxious temperature. Based on this observation, we suggest that noxious heat may directly activate specific guanylyl cyclases such as GCY-12, whereas a rather slower G protein signaling pathway is used in sensing ambient temperature.

Numerous animal studies have shown that cGMP essentially contributes to the sensitization of both inflammatory and neuropathic pain [46]. Although the role of CNG channels in pain sensation as downstream target of cGMP has not been elucidated yet, CNG channel subunits are expressed in vertebrate dorsal root ganglia (DRG), where the nociceptors locate [46]. To our knowledge, our study provides the first evidence of CNG channels functioning in the sensation of noxious temperature. Given that CNG and TRPV channels are expressed in different cells and act in cell-autonomous manner, we propose that both have distinct roles in the heat avoidance response. It is currently not possible to decide whether they function independently of one another, since we observed that *tax-2;tax-4* double mutants did not further decrease the Tav behavior in the head of *ocr-2osm-9* mutant animals (Table S6). This may either indicate a redundant function of OCR-2 and OSM-9 in the FLP sensory neurons or a more complex interaction between these channels and their sensory neurons in the noxious heat response.

Recent studies indicate that in mammals at least two different mechanisms are present for detecting noxious heat, TRPV1-dependent and TRPV1-independent mechanisms. Although the TRPV1-dependent mechanism has been studied in great detail, the TRPV1-independent mechanisms are still unknown [2], [3], [4], [47]. Similarly, we show that in *C. elegans* two independent mechanisms are involved in the sensation of noxious heat. One of them uses the TRPV-channels OSM-9/OCR-2 in the FLP and PHC thermonociceptors and the other the CNG-channel complex TAX-2/TAX-4 in the AFD neurons (Figure 6C). Future work will show whether the cGMP/CNG pathway involves neuropeptide signaling that was suggested as a TRPV1-independent mechanism [8], [44]. Therefore, the thermal avoidance response is mediated by a carefully backed-up system by using different cell types and at least two signaling mechanisms. This redundancy may be necessary for robust and reliable responses upon exposure to life-threatening stimuli like heat. It will be important to elucidate the crosstalk between sensory AFD, FLP and PHC sensory neurons and the heat-activated proteins involved in thermonociception.

## Materials and Methods

### Ethics Statement

Experimental work on the invertebrate animal *Caenorhabditis elegans* does not require the approval of a named review board institution or ethics committee in Germany. Studies on human or vertebrate animals were not performed by us in this study.

### Strains

Nematodes were grown at 20°C with abundant food using standard methods [48]. The *tax-4(p678)* and *tax-2(p671)* mutants and all TRPV1 mutants were backcrossed four times after acquisition before performing experiments. All the other mutants (see Table S10) used were backcrossed two times if they had not been backcrossed before.

### Behavioral and statistical analysis

The thermal avoidance assays were performed using a monochromatic laser diode that emits at 685±0.5 nm as described before [8], with the modification that the laser focus was presented for 10 seconds each at the tip of the nose or the tip of the tail of the animals for stimulation. The initial response upon stimulation was scored as a positive Tav response. The positive head Tav response is a withdrawal reaction, whereas the positive tail Tav response is either an accelerated forward movement of forward moving animals or an initiated backward movement of resting animals.

The nose touch response was tested as described previously by placing an eyelash in the path of an animal moving forward [6].

Statistical analysis was carried out using the GraphPad Prism 4 software. P values were generated by one-way ANOVA using the Tukey-test and were shown in the supplementary tables.

### 2-photon neuron ablation

Individual neurons were identified by GFP-labeling (for strains see Table S7). Neuron ablation was performed essentially as described previously [49]. Animals were mounted and paralyzed on 4% (in M9 buffer containing 0.6 mM levamisole) agarose pads within 2 µl M9 buffer. Neurons from L2-stage animals were ablated with a 2-photon-laser (wavelength 730 nm) microbeam focused through the C-Apochromat 63×/1.2w corr objective of a confocal microscope Zeiss LSM 510 Meta NLO. Ablated animals were tested for their Tav response after recovery for at least 2 days at 20°C (to let them grow up to young adults). Control animals of the same genotype were exposed to the same treatment except for neuron-laser-ablation. The success of the neuron ablation was monitored by the disappearance of GFP in the neurons instantly after the ablation as well as after the behavior test.

### Molecular biology and germline transformation

The plasmids with full-length genomic DNA *osm-9::GFP5* [50], *ocr-2::GFP* [15], *tax-4::GFP* [11] and *tax-2::GFP* [51] were used for rescue. To generate rescue transgenic lines, the plasmids (25 ng/µl) were microinjected together with the co-injection marker *Pmyo-2::mCherry* (20 ng/µl) in the respective mutants.

We obtained the *ocr-2*, *osm-9* and *tax-2* cDNA from C. Bargmann and *tax-4* cDNA from I. Mori. *Pgcy-8::tax-2::GFP* and *Pgcy-8::tax-4::GFP* were generated by cloning the cDNA with KpnI, XmaI sites downstream of a *gcy-8* promoter in pPD95.75. *Podr-4::tax-2::GFP* and *Podr-4::tax-4::GFP* were generated by cloning the *odr-4* promoter (4.6 kb upstream the coding region) before the cDNA with SphI, XmaI to replace the *gcy-8* promoter. *Pmec-3::ocr-2::GFP* and *Pmec-3::osm-9::GFP* were generated by cloning the cDNA with XhoI, SmaI sites downstream of the *mec-3* promoter in EGFP-N1. Transgenic strains were generated by injecting the mutants with the rescue construct (25 ng/µl) and the co-injection marker *Pmyo-2::mCherry* (20 ng/µl) or *Punc-122::RFP* (20 ng/µl). For rescue experiments, at least two transgenic lines carrying arrays were analyzed and data from a representative array were shown.

The plasmids *Pgcy-8::DT-A* and *Podr-3::DT-A* (*Diphtheria* toxin A) were generated by cloning DT-A cDNA with XmaI, KpnI sites downstream of the *gcy-8* or *odr-3* promoter. To eliminate neurons genetically, two transgenic lines carrying the DT-A construct (1 ng/µl) and the co-injection marker *Pmyo-2::mCherry* (20 ng/µl) were generated.

The plasmids containing *YC2.12* (PKDK153) and *YC4.12* cDNA were obtained from I. Mori. *Pgcy-8::YC4.12* was generated by cloning the *gcy-8* promoter (3 kb upstream) with SbfI and Acc65I sites in the vector. For unknown reasons, we could not obtain transgenic lines with *Pgcy-8::YC4.12*. F1 animals were tested for their calcium imaging instead. *Pmec-3::YC2.12* was generated by cloning the *mec-3* promoter (2 kb upstream and 1.3 kb genomic sequence) with XmaI, NheI sites in PKDK153. *Pida-1::YC2.12* was cloned by inserting the *ida-1* promoter (2.4 kb upstream) with FseI and AscI sites in the vector. Transgenic strains were generated by injecting N2 animals with the expression construct (100 ng/µl) and the co-injection marker *Punc-122::RFP* (20 ng/µl). All the generated plasmids were shown in Table S8 and the transgenic strains used in this study were shown in Table S9.

### Calcium imaging and data analysis

Single young adult worms were mounted as described before [52] on a thin 2% agarose pad with small amounts of surgical N-butyl(2)-cyanoacrylate (Histoacryl) glue. The agarose pad was made on a large cover-slip. The glue was delivered through a drawn glass capillary tube operated by mouth. Gluing the tip of the animal's head was avoided. The worm was then covered with a smaller cover-slip. The noxious temperature stimuli were generated using a standard chip sized ceramic SMD0805 electrical resistor (size 2×1.25×0.5 mm^3^) placed on the smaller cover slip. 0.2 µl of mineral oil were used to displace the air between the resistor and the glass. An ATMEL AVR ATMEGA88 microcontroller served as a triggerable electronic timer to produce local heat pulses of 190 mW and adjustable duration. The conditions of the laser-based Tav assay on plate were reproduced by a typically 2 seconds long heating, which generated a local temperature peak of ∼38°C with a temperature increase of 8°C/s, and which also induced slight withdrawal response of the animal's head. General room temperature was kept at ∼22°C during optical recording. Due to lack of a sensitized CCD camera system, a Nikon A1 confocal laser scanning microscope and a Nikon CFI Plan Apochromat VC 60×WI N.A. 1.20 objective was used to record the calcium dynamics of a single neuron for 5 seconds before the stimulus and 5 seconds after the beginning of the stimulus. A field of 64×64 pixel was recorded with 4 frames per second. Excitation wavelength was 458 nm, emitted light was collected at 465–500 nm (CFP) and 525–555 nm (YFP) respectively. Regions of interest around the cell body of the neuron were defined by hand and adjusted for each image frame accordingly because of the motion of the sample. YFP/CFP ratios were determined using Nikon NIS-Elements software version 3.10. The average of the 5 s pre-stimulus baseline ratio was set as R_0_. The percentage of the fluorescence ratios relative to R_0_ were calculated as [(R_t_−R_0_)/R_0_]*100%, where R_t_ means a ratio at the time t. The average magnitude of the ratio change (maximum ratio-minimum ratio) from more than 4 animals was plotted as Box & Whisker Plots using the GraphPad Prism 4 software. P values were generated by one-way ANOVA using the Tukey-test.

## Supporting Information

Figure S1 **Expression of DT-A under the control of AFD specific** ***gcy-8*** **promoter successfully ablated the AFD neurons.** (a) The fluorescence micrograph of an animal (strain BR5256) carrying GFP reporter in both AFD and FLP in the head. The AFD neurons are indicated by stars and the FLP neurons by arrowheads. (b) The elimination of GFP reporter in the AFD neurons in strain BR5634 carrying DT-A in AFD indicated the successful ablation of AFD. Residual GFP in the necrotic AFD neuron is indicated by a star. Images (40-fold magnification) are confocal Z series projected into a single plane. Scale bars represent 10 µm. The anterior part of the animal is oriented to the left.
(TIF)

Figure S2 **Representative traces of AFD, FLP, and PHC neuron responses to the noxious heat stimulus.** Four representative traces of AFD, FLP, and PHC neuron responses to the noxious heat stimulus are presented. Heating was turned on at the 5th second after recording and reached 38°C at the 7th second. The response was plotted as fractional YFP/CFP ratio change over baseline.
(TIF)

Figure S3 **Dendrogram of the TRPV subfamily of TRP-related ion channels.** The *C. elegans* gene family was identified by a Hidden Markov Model (HMM) search in WormPep, aligned using ClustalX, and a neighbor joining tree was calculated with ClustalX.
(DOCX)

Table S1 **The AFD and FLP neurons mediate the Tav response in the head, while the PHC neurons are essential for the Tav response in the tail of** ***C. elegans*** **.** Values reported are mean % ± SD %; ND: not determined; n^A^ denotes number of animals tested, each animal was tested at least 4 times for the Tav response; *p* ^B^ values are compared to respective mock treated animals for the Tav response in the head; *p* ^C^ values are compared to respective mock treated animals for the Tav response in the tail.
(DOCX)

Table S2 **Transgenic animals carrying DT-A in ASH fail to avoid touch to the nose.** Values reported are mean % ± SD %; n^A^ denotes number of animals tested, each animal was tested at least 4 times for the nose touch response; *p* ^B^ values are compared to the wild-type animals.
(DOCX)

Table S3 **Mutant animals with developmental defects in the AFD, FLP and PHC neurons failed to avoid noxious temperature stimuli at the head or tail of** ***C. elegans*** **.** Values reported are mean % ± SD %; n^A^ denotes number of animals tested, 3–17 independent assays were performed; *p* ^B^ values are compared to wild-type animals for the Tav response in the head; *p* ^C^ values are compared to wild-type animals for the Tav response in the tail; ^E^ *deg-1(u38)* mutants showed only initiated backward movement and no forward movement in the Tav response in the posterior part.
(DOCX)

Table S4 **The Tav responses in transgenic animals expressing cameleon for calcium imaging.** Values reported are mean % ± SD %; n^A^ denotes number of animals tested, 3–17 independent assays were performed; *p* ^B^ values are compared to wild-type animals for the Tav response in the head; *p* ^C^ values are compared to wild-type animals for the Tav response in the tail.
(DOCX)

Table S5 **OCR-2 and OSM-9 are required in the FLP and PHC neurons of** ***C. elegans*** **for the Tav response.** Values reported are mean % ± SD %; n^A^ denotes number of animals tested, 3–17 independent assays were performed; *p* ^B^ values are compared to wild-type animals for the Tav response in the head; *p* ^C^ values are compared to wild-type animals for the Tav response in the tail; *p* ^D^ values are compared to *ocr-2(vs29)osm-9(ky10)* for the Tav response in the head; *p* ^E^ values are compared to *ocr-2(vs29)osm-9(ky10)* for the Tav response in the tail.
(DOCX)

Table S6 **TAX-2 and TAX-4 are required in the AFD neurons in the Tav response in the anterior part of** ***C. elegans*** **.** Values reported are mean % ± SD %; n^A^ denotes number of animals tested, 3–17 independent assays were performed; ND: not determined; *p* ^B^ values are compared to wild-type animals for the Tav response in the head; *p* ^C^ values are compared to wild-type animals for the Tav response in the tail; *p* ^D^ values are compared to *tax-4(p678);byEx925[myo-2::mCherry]*; *p* ^E^ values are compared to *tax-2(p671);byEx925[myo-2::mCherry]*.
(DOCX)

Table S7 **Strains used for laser-ablation to label neurons.**
(DOCX)

Table S8 **Plasmids constructed in this study.**
(DOCX)

Table S9 **Transgenic strains used in this study.**
(DOCX)

Table S10 **References for mutant strains.**
(DOCX)

## References

[1] Caterina MJ, Rosen TA, Tominaga M, Brake AJ, Julius D (1999). A capsaicin-receptor homologue with a high threshold for noxious heat.. Nature.

[2] Caterina MJ, Leffler A, Malmberg AB, Martin WJ, Trafton J (2000). Impaired nociception and pain sensation in mice lacking the capsaicin receptor.. Science.

[3] Woodbury CJ, Zwick M, Wang S, Lawson JJ, Caterina MJ (2004). Nociceptors lacking TRPV1 and TRPV2 have normal heat responses.. J Neurosci.

[4] Basbaum AI, Bautista DM, Scherrer G, Julius D (2009). Cellular and molecular mechanisms of pain.. Cell.

[5] Chang AJ, Chronis N, Karow DS, Marletta MA, Bargmann CI (2006). A distributed chemosensory circuit for oxygen preference in C. elegans.. PLoS Biol.

[6] Kaplan JM, Horvitz HR (1993). A dual mechanosensory and chemosensory neuron in Caenorhabditis elegans.. Proc Natl Acad Sci U S A.

[7] Bargmann CI (2006). Chemosensation in C. elegans.. Worm Book.

[8] Wittenburg N, Baumeister R (1999). Thermal avoidance in Caenorhabditis elegans: an approach to the study of nociception.. Proc Natl Acad Sci U S A.

[9] Ward A, Liu J, Feng Z, Xu XZ (2008). Light-sensitive neurons and channels mediate phototaxis in C. elegans.. Nat Neurosci.

[10] Kimura KD, Miyawaki A, Matsumoto K, Mori I (2004). The C. elegans thermosensory neuron AFD responds to warming.. Curr Biol.

[11] Komatsu H, Mori I, Rhee JS, Akaike N, Ohshima Y (1996). Mutations in a cyclic nucleotide-gated channel lead to abnormal thermosensation and chemosensation in C. elegans.. Neuron.

[12] Komatsu H, Jin YH, L'Etoile N, Mori I, Bargmann CI (1999). Functional reconstitution of a heteromeric cyclic nucleotide-gated channel of Caenorhabditis elegans in cultured cells.. Brain Res.

[13] Wei JY, Roy DS, Leconte L, Barnstable CJ (1998). Molecular and pharmacological analysis of cyclic nucleotide-gated channel function in the central nervous system.. Prog Neurobiol.

[14] Bargmann CI, Mori I (1997). Chemotaxis and Thermotaxis.

[15] Tobin D, Madsen D, Kahn-Kirby A, Peckol E, Moulder G (2002). Combinatorial expression of TRPV channel proteins defines their sensory functions and subcellular localization in C. elegans neurons.. Neuron.

[16] Woolf CJ, Ma Q (2007). Nociceptors–noxious stimulus detectors.. Neuron.

[17] Albeg A, Smith CJ, Chatzigeorgiou M, Feitelson DG, Hall DH (2011). C. elegans multi-dendritic sensory neurons: morphology and function.. Mol Cell Neurosci.

[18] Roayaie K, Crump JG, Sagasti A, Bargmann CI (1998). The G alpha protein ODR-3 mediates olfactory and nociceptive function and controls cilium morphogenesis in C. elegans olfactory neurons.. Neuron.

[19] Kuhara A, Okumura M, Kimata T, Tanizawa Y, Takano R (2008). Temperature sensing by an olfactory neuron in a circuit controlling behavior of C. elegans.. Science.

[20] Hobert O, Mori I, Yamashita Y, Honda H, Ohshima Y (1997). Regulation of interneuron function in the C. elegans thermoregulatory pathway by the ttx-3 LIM homeobox gene.. Neuron.

[21] White JG, Southgate E, Thomson JN, Brenner S (1986). The Structure of the Nervous System of the Nematode Caenorhabditis elegans.. Philosophical Transactions of the Royal Society of London Series B, Biological Sciences.

[22] Finney M, Ruvkun G (1990). The unc-86 gene product couples cell lineage and cell identity in C. elegans.. Cell.

[23] Baumeister R, Liu Y, Ruvkun G (1996). Lineage-specific regulators couple cell lineage asymmetry to the transcription of the Caenorhabditis elegans POU gene unc-86 during neurogenesis.. Genes Dev.

[24] Hall DH, Russell RL (1991). The posterior nervous system of the nematode Caenorhabditis elegans: serial reconstruction of identified neurons and complete pattern of synaptic interactions.. J Neurosci.

[25] Chalfie M, Wolinsky E (1990). The identification and suppression of inherited neurodegeneration in Caenorhabditis elegans.. Nature.

[26] Miyawaki A, Llopis J, Heim R, McCaffery JM, Adams JA (1997). Fluorescent indicators for Ca2+ based on green fluorescent proteins and calmodulin.. Nature.

[27] Jurado P, Kodama E, Tanizawa Y, Mori I (2010). Distinct thermal migration behaviors in response to different thermal gradients in Caenorhabditis elegans.. Genes Brain Behav.

[28] Kerr R, Lev-Ram V, Baird G, Vincent P, Tsien RY (2000). Optical imaging of calcium transients in neurons and pharyngeal muscle of C. elegans.. Neuron.

[29] Chatzigeorgiou M, Yoo S, Watson JD, Lee WH, Spencer WC (2010). Specific roles for DEG/ENaC and TRP channels in touch and thermosensation in C. elegans nociceptors.. Nat Neurosci.

[30] Madison JM, Nurrish S, Kaplan JM (2005). UNC-13 interaction with syntaxin is required for synaptic transmission.. Curr Biol.

[31] Speese S, Petrie M, Schuske K, Ailion M, Ann K (2007). UNC-31 (CAPS) is required for dense-core vesicle but not synaptic vesicle exocytosis in Caenorhabditis elegans.. J Neurosci.

[32] Jose AM, Bany IA, Chase DL, Koelle MR (2007). A specific subset of transient receptor potential vanilloid-type channel subunits in Caenorhabditis elegans endocrine cells function as mixed heteromers to promote neurotransmitter release.. Genetics.

[33] Way JC, Chalfie M (1989). The mec-3 gene of Caenorhabditis elegans requires its own product for maintained expression and is expressed in three neuronal cell types.. Genes Dev.

[34] Cho SW, Choi KY, Park CS (2004). A new putative cyclic nucleotide-gated channel gene, cng-3, is critical for thermotolerance in Caenorhabditis elegans.. Biochem Biophys Res Commun.

[35] Cho SW, Cho JH, Song HO, Park CS (2005). Identification and characterization of a putative cyclic nucleotide-gated channel, CNG-1, in C. elegans.. Mol Cells.

[36] Coburn CM, Bargmann CI (1996). A putative cyclic nucleotide-gated channel is required for sensory development and function in C. elegans.. Neuron.

[37] Dwyer ND, Troemel ER, Sengupta P, Bargmann CI (1998). Odorant receptor localization to olfactory cilia is mediated by ODR-4, a novel membrane-associated protein.. Cell.

[38] Inada H, Ito H, Satterlee J, Sengupta P, Matsumoto K (2006). Identification of guanylyl cyclases that function in thermosensory neurons of Caenorhabditis elegans.. Genetics.

[39] Perkins LA, Hedgecock EM, Thomson JN, Culotti JG (1986). Mutant sensory cilia in the nematode Caenorhabditis elegans.. Dev Biol.

[40] Bretscher AJ, Kodama-Namba E, Busch KE, Murphy RJ, Soltesz Z (2011). Temperature, Oxygen, and Salt-Sensing Neurons in C. elegans Are Carbon Dioxide Sensors that Control Avoidance Behavior.. Neuron.

[41] Hwang RY, Zhong L, Xu Y, Johnson T, Zhang F (2007). Nociceptive neurons protect Drosophila larvae from parasitoid wasps.. Curr Biol.

[42] Wicks SR, Rankin CH (1995). Integration of mechanosensory stimuli in Caenorhabditis elegans.. J Neurosci.

[43] Bennett MV, Zukin RS (2004). Electrical coupling and neuronal synchronization in the Mammalian brain.. Neuron.

[44] Glauser DA, Chen WC, Agin R, Macinnis BL, Hellman AB (2011). Heat Avoidance is Regulated by Transient Receptor Potential (TRP) Channels and a Neuropeptide Signaling Pathway in Caenorhabditis elegans.. Genetics.

[45] Hilliard MA, Bargmann CI, Bazzicalupo P (2002). C. elegans responds to chemical repellents by integrating sensory inputs from the head and the tail.. Curr Biol.

[46] Schmidtko A, Tegeder I, Geisslinger G (2009). No NO, no pain? The role of nitric oxide and cGMP in spinal pain processing.. Trends Neurosci.

[47] Lawson JJ, McIlwrath SL, Woodbury CJ, Davis BM, Koerber HR (2008). TRPV1 unlike TRPV2 is restricted to a subset of mechanically insensitive cutaneous nociceptors responding to heat.. J Pain.

[48] Brenner S (1974). The genetics of Caenorhabditis elegans.. Genetics.

[49] Bargmann CI, Avery L (1995). Laser killing of cells in Caenorhabditis elegans.. Methods Cell Biol.

[50] Colbert HA, Smith TL, Bargmann CI (1997). OSM-9, a novel protein with structural similarity to channels, is required for olfaction, mechanosensation, and olfactory adaptation in Caenorhabditis elegans.. J Neurosci.

[51] Coburn CM, Mori I, Ohshima Y, Bargmann CI (1998). A cyclic nucleotide-gated channel inhibits sensory axon outgrowth in larval and adult Caenorhabditis elegans: a distinct pathway for maintenance of sensory axon structure.. Development.

[52] Hilliard MA, Apicella AJ, Kerr R, Suzuki H, Bazzicalupo P (2005). In vivo imaging of C. elegans ASH neurons: cellular response and adaptation to chemical repellents.. EMBO J.

