# Supplementary figures and images for: Temperature- and Touch-Sensitive Neurons Couple CNG and TRPV Channel Activities to Control Heat Avoidance in Caenorhabditis elegans
Source: PLoS One. 2012 Mar 20;7(3):e32360. doi: 10.1371/journal.pone.0032360 (PMC3308950; doi:10.1371/journal.pone.0032360)

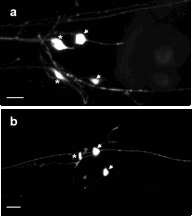

Supplement: Figure S1 — Expression of DT-A under the control of AFD specific gcy-8 promoter successfully ablated the AFD neurons. (a) The fluorescence micrograph of an animal (strain BR5256) carrying GFP reporter in both AFD and FLP in the head. The AFD neurons are indicated by stars and the FLP neurons by arrowheads. (b) The elimination of GFP reporter in the AFD neurons in strain BR5634 carrying DT-A in AFD indicated the successful ablation of AFD. Residual GFP in the necrotic AFD neuron is indicated by a star. Images (40-fold magnification) are confocal Z series projected into a single plane. Scale bars represent 10 µm. The anterior part of the animal is oriented to the left. (TIF) [file pone.0032360.s001.tif]

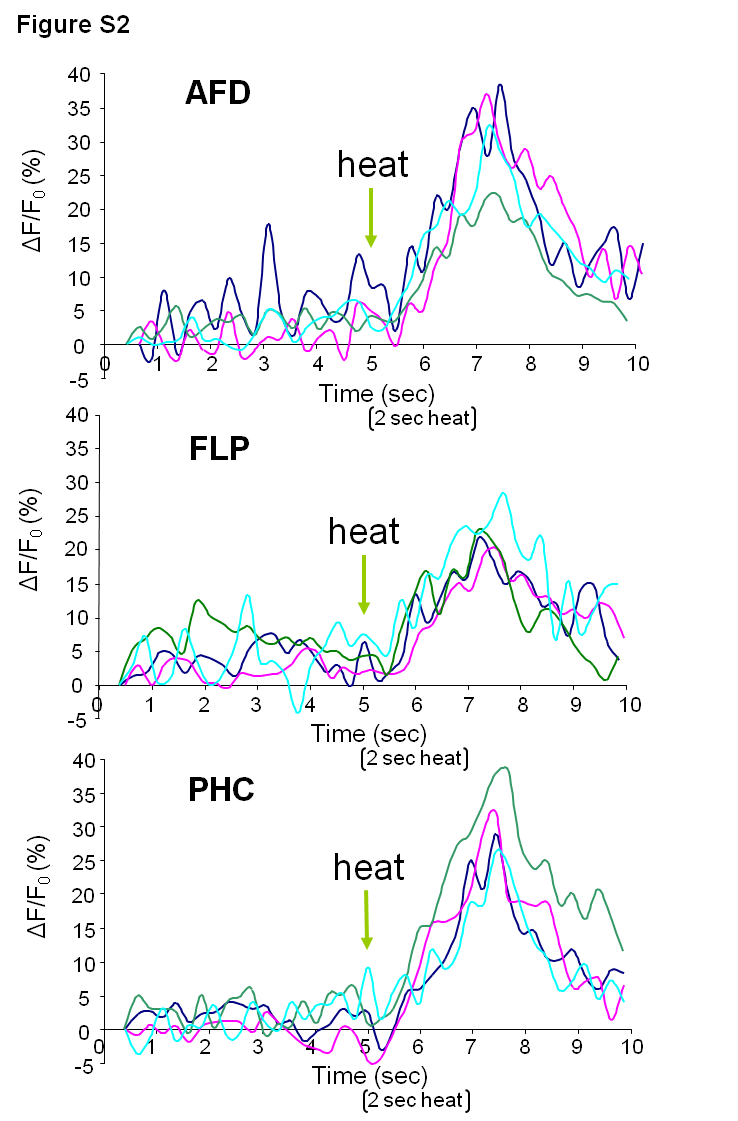

Supplement: Figure S2 — Representative traces of AFD, FLP, and PHC neuron responses to the noxious heat stimulus. Four representative traces of AFD, FLP, and PHC neuron responses to the noxious heat stimulus are presented. Heating was turned on at the 5th second after recording and reached 38°C at the 7th second. The response was plotted as fractional YFP/CFP ratio change over baseline. (TIF) [file pone.0032360.s002.tif]
