# Supplementary material for: Temperature- and Touch-Sensitive Neurons Couple CNG and TRPV Channel Activities to Control Heat Avoidance in Caenorhabditis elegans
Source: PLoS One. 2012 Mar 20;7(3):e32360. doi: 10.1371/journal.pone.0032360 (PMC3308950; doi:10.1371/journal.pone.0032360)
Supplement: Figure S3 — Dendrogram of the TRPV subfamily of TRP-related ion channels. The C. elegans gene family was identified by a Hidden Markov Model (HMM) search in WormPep, aligned using ClustalX, and a neighbor joining tree was calculated with ClustalX. (DOCX) [file pone.0032360.s003.docx]

**Figure S3. Dendrogram of the TRPV subfamily of TRP‑related ion channels**


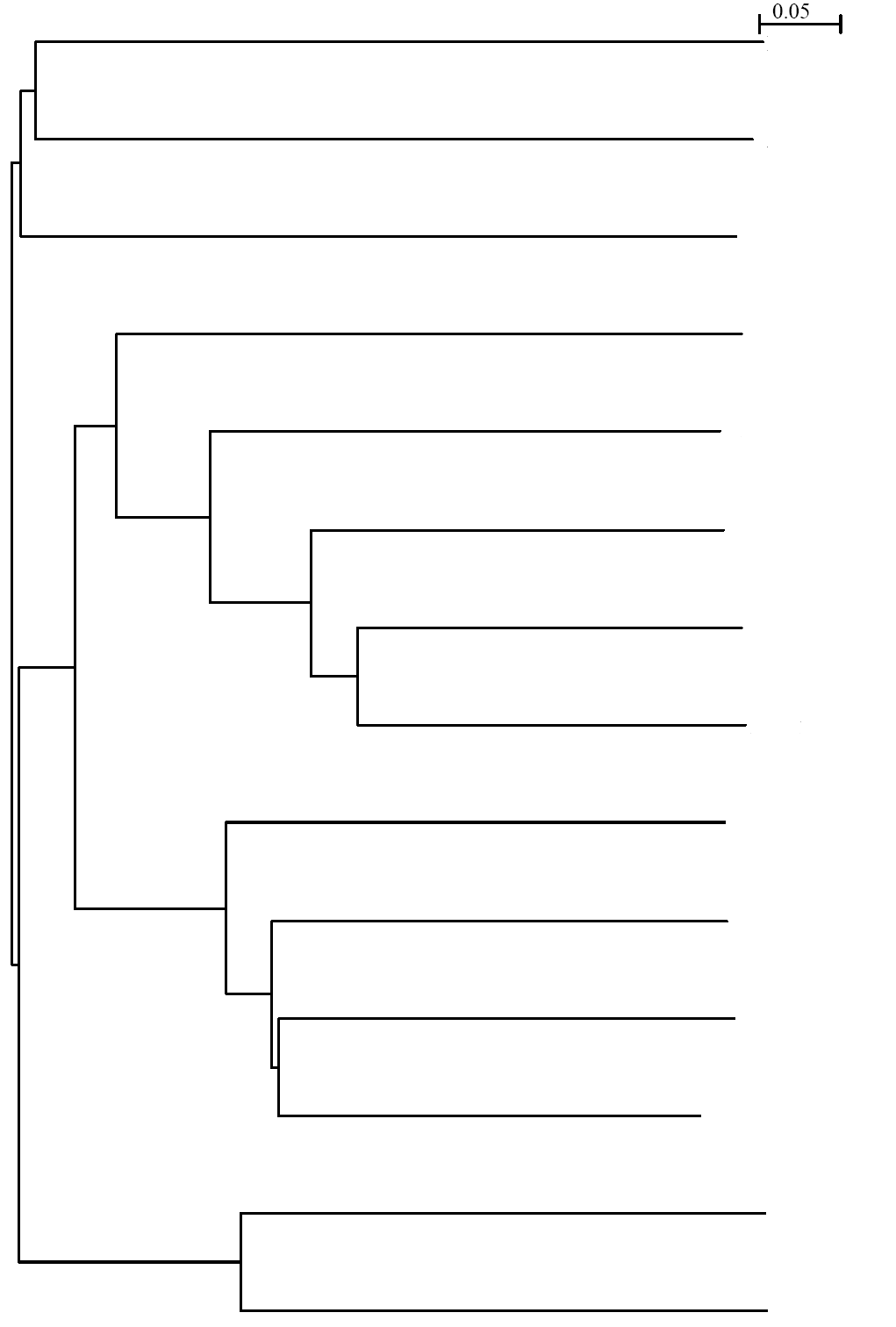


*unc*

*-*

*44*

*trpa*

*-*

*1*

*trp*

*-*

*4*

*osm*

*-*

*9*

*ocr*

*-*

*4*

*ocr*

*-*

*3*

*ocr*

*-*

*2*

*ocr*

*-*

*1*

*trp*

*-*

*2*

*trp*

*-*

*1*

*-*

*44*

*trpa*

*trp*

*-*

*osm*

*-*

*9*

*ocr*

*4*

*ocr*

*-*

*3*

*ocr*

*2*

*ocr*

*-*

*-*

*trp*

*-*

TRPV3

TRPV2

TRPV4

TRPV1
