# Supplementary material for: Temperature- and Touch-Sensitive Neurons Couple CNG and TRPV Channel Activities to Control Heat Avoidance in Caenorhabditis elegans
Source: PLoS One. 2012 Mar 20;7(3):e32360. doi: 10.1371/journal.pone.0032360 (PMC3308950; doi:10.1371/journal.pone.0032360)
Supplement: Table S1 — The AFD and FLP neurons mediate the Tav response in the head, while the PHC neurons are essential for the Tav response in the tail of C. elegans . Values reported are mean % ± SD %; ND: not determined; nA denotes number of animals tested, each animal was tested at least 4 times for the Tav response; p B values are compared to respective mock treated animals for the Tav response in the head; p C values are compared to respective mock treated animals for the Tav response in the tail. (DOCX) [file pone.0032360.s004.docx]

Table S1. The AFD and FLP neurons mediate the Tav response in the head, while the PHC neurons are essential for the Tav response in the tail of *C. elegans*

| **neurons ablated** | **Tav response in the head** | **Tav response in the tail** | **n ^A^** | ***p* value^B^** | ***p* value^C^** |
| --- | --- | --- | --- | --- | --- |
| **1. AFD and FLP** |  |  |  |  |  |
| AFD‑ablated animals | 40.2 ± 7.1 | ND | 23 | <0.001 |  |
| mock‑ablated (AFD) | 88.5 ± 1.6 | ND | 44 |  |  |
| BR5256;*byEx851[Pgcy-8::DTA;myo-2::mCherry]* | 42.3 ± 16.3 | 61.2 ± 3.2 | 83 | <0.001 | >0.05 |
| BR5256;*byEx852[Pgcy-8::DTA;myo-2::mCherry]* | 36.6 ± 7.9 | 64.8 ± 4.9 | 108 | <0.001 | >0.05 |
| N2;*byEx925[myo-2::mCherry]* | 89.9 ± 3.8 | 68.0 ± 10 | 96 |  |  |
| N2;*byEx1025[Podr‑3::DTA;Podr‑4::GFP;myo‑2::mCherry]* | 89.3 ± 5.7 | 64.2 ± 2.1 | 57 | >0.05 | >0.05 |
| FLP‑ablated animals | 61.1 ± 9.6 | ND | 11 | <0.01 |  |
| mock‑ablated (FLP) | 87.8 ± 0.3 | ND | 17 |  |  |
| AFD,FLP‑ablated animals | 7.8 ± 9.7 | 65.0 ± 49.5 | 19 | <0.001 | >0.05 |
| mock‑ablated (AFD,FLP) | 100 | 88.9 ± 19.2 | 17 |  |  |
| **2. AIY and AIB** |  |  |  |  |  |
| AIY‑ablated animals | 100 | ND | 8 | >0.05 |  |
| mock‑ablated (AIY) | 100 | ND | 14 |  |  |
| AIB‑ablated animals | 33.3 | ND | 13 | <0.001 |  |
| mock‑ablated (AIB) | 93.8 ± 8.8 | ND | 9 |  |  |
| AFD,AIB‑ablated animals | 32.5 ± 10.6 | 55 ± 7.1 | 9 | <0.001 | >0.05 |
| mock‑ablated (AFD,AIB) | 100 | 50 | 8 |  |  |
| **3. amphid** |  |  |  |  |  |
| ASH‑ablated animals | 87.5 ± 12.5 | ND | 21 | >0.05 |  |
| mock‑ablated (ASH) | 76.4 ± 1.4 | ND | 13 |  |  |
| AWA‑ablated animals | 100 | ND | 9 | >0.05 |  |
| mock‑ablated (AWA) | 88.9 | ND | 7 |  |  |
| ADF‑ablated animals | 100 | ND | 6 | >0.05 |  |
| mock‑ablated (ADF) | 100 | ND | 7 |  |  |
| ADL‑ablated animals | 100 | ND | 12 | >0.05 |  |
| mock‑ablated (ADL) | 100 | ND | 12 |  |  |
| AWB‑ablated animals | 95.0 ± 7.1 | ND | 16 | >0.05 |  |
| mock‑ablated (AWB) | 100 | ND | 18 |  |  |
| BAG‑ablated animals | 100 | ND | 9 | >0.05 |  |
| mock‑ablated (BAG) | 100 | ND | 7 |  |  |
| **4. ALM, AVM, PLM, PVM** |  |  |  |  |  |
| neuron‑ablated animals | 100 | 66.7 | 9 | >0.05 | >0.05 |
| mock‑ablated animals | 100 | 70.9 ± 5.9 | 10 |  |  |
| PVD‑ablated animals | 100 | 75 | 7 | >0.05 | >0.05 |
| mock‑ablated (PVD) | 100 | 71.4 | 8 |  |  |
| **5. URX, AQR, PQR** |  |  |  |  |  |
| wild‑type animals | 95.2 ± 2.0 | 68.1 ± 6.0 | 628 |  |  |
| BR4983 N2;*quaIs2241[Pgcy-36::egl-1;Pgcy-35::gfp;lin-15(+)]* | 94.1 ± 1.7 | 62.2 ± 9.2 | 187 | >0.05 | >0.05 |
| **6. phasmid** |  |  |  |  |  |
| PHC‑ablated animals | 100 | 10 ± 11.6 | 21 | >0.05 | <0.001 |
| mock‑ablated (PHC) | 100 | 70.9 ± 5.9 | 22 |  |  |
| PHA,PHB‑ablated animals | 100 | 63.4 ± 4.7 | 12 | >0.05 | >0.05 |
| mock‑ablated (PHA,PHB) | 100 | 66.7 | 6 |  |  |

Values reported are mean % ± SD %

ND: not determined

n^A^ denotes number of animals tested, each animal was tested at least 4 times for the Tav response.

*p*^B^ values are compared to respective mock treated animals for the Tav response in the head.

*p*^C^ values are compared to respective mock treated animals for the Tav response in the tail.
