# Supplementary material for: Temperature- and Touch-Sensitive Neurons Couple CNG and TRPV Channel Activities to Control Heat Avoidance in Caenorhabditis elegans
Source: PLoS One. 2012 Mar 20;7(3):e32360. doi: 10.1371/journal.pone.0032360 (PMC3308950; doi:10.1371/journal.pone.0032360)
Supplement: Table S2 — Transgenic animals carrying DT-A in ASH fail to avoid touch to the nose. Values reported are mean % ± SD %; nA denotes number of animals tested, each animal was tested at least 4 times for the nose touch response; p B values are compared to the wild-type animals. (DOCX) [file pone.0032360.s005.docx]

Table S2. Transgenic animals carrying DT-A in ASH fail to avoid touch to the nose

| **Genotype** | **Avoidance, % responding** | **n^A^** | ***p* value^B^** |
| --- | --- | --- | --- |
| wild‑type | 95.0 ± 7.1 | 25 |  |
| N2;*byEx1025[Podr-3::DTA;Podr-4::gfp;myo-2::mCherry]* | 36.1 ± 19.1 | 32 | <0.01 |
| N2;*byEx1026[Podr-3::DTA;Podr-4::gfp;myo-2::mCherry]* | 36.9 ± 4.7 | 38 | <0.01 |

Values reported are mean % ± SD %

n^A^ denotes number of animals tested, each animal was tested at least 4 times for the nose touch response.

*p*^B^ values are compared to the wild-type animals.
