# Supplementary material for: Temperature- and Touch-Sensitive Neurons Couple CNG and TRPV Channel Activities to Control Heat Avoidance in Caenorhabditis elegans
Source: PLoS One. 2012 Mar 20;7(3):e32360. doi: 10.1371/journal.pone.0032360 (PMC3308950; doi:10.1371/journal.pone.0032360)
Supplement: Table S3 — Mutant animals with developmental defects in the AFD, FLP and PHC neurons failed to avoid noxious temperature stimuli at the head or tail of C. elegans . Values reported are mean % ± SD %; nA denotes number of animals tested, 3–17 independent assays were performed; p B values are compared to wild-type animals for the Tav response in the head; p C values are compared to wild-type animals for the Tav response in the tail; E deg-1(u38) mutants showed only initiated backward movement and no forward movement in the Tav response in the posterior part. (DOCX) [file pone.0032360.s006.docx]

Table S3. Mutant animals with developmental defects in the AFD, FLP and PHC neurons faile to avoid noxious temperature stimuli at the head or tail of *C. elegans*

| **Genotype** | **Tav response in the head** | **Tav response in the tail** | **n^A^** | ***p* value^B^** | ***p* value^C^** |
| --- | --- | --- | --- | --- | --- |
| wild‑type | 95.2 ± 2.0 | 68.1 ± 6.0 | 628 |  |  |
| *sem-4(n1378)* | 95.0 ± 7.1 | 17.4 ± 12.4 | 179 | >0.05 | <0.001 |
| *unc-86(n846)* | 80.7 ± 13.9 | 13.8 ± 7.7 | 197 | <0.01 | <0.001 |
| *deg-1(u38)* | 98.2 ± 1.8 | 15.8 ± 2.3^E^ | 130 | >0.05 | <0.001 |
| *ttx-3(ot22)* | 93.5 ± 4.6 | 65.1 ± 5.6 | 88 | >0.05 | >0.05 |
| *ttx-3(mg158)* | 92.7 ± 2.1 | 69.9 ± 6.1 | 146 | >0.05 | >0.05 |

Values reported are mean % ± SD %

n^A^ denotes number of animals tested, 3-17 independent assays were performed.

*p^B^* values are compared to wild-type animals for the Tav response in the head.

*p*^C^ values are compared to wild-type animals for the Tav response in the tail.

^E^ *deg-1(u38)* mutants showed only initiated backward movement and no forward movement in the Tav response in the posterior part.
