# Supplementary material for: Temperature- and Touch-Sensitive Neurons Couple CNG and TRPV Channel Activities to Control Heat Avoidance in Caenorhabditis elegans
Source: PLoS One. 2012 Mar 20;7(3):e32360. doi: 10.1371/journal.pone.0032360 (PMC3308950; doi:10.1371/journal.pone.0032360)
Supplement: Table S4 — The Tav responses in transgenic animals expressing cameleon for calcium imaging. Values reported are mean % ± SD %; nA denotes number of animals tested, 3–17 independent assays were performed; p B values are compared to wild-type animals for the Tav response in the head; p C values are compared to wild-type animals for the Tav response in the tail. (DOCX) [file pone.0032360.s007.docx]

**Table S4. The Tav responses in transgenic animals expressing cameleon for calcium imaging**

| **Genotype** | **Tav response in the head** | **Tav response in the tail** | **n ^A^** | ***p* value^B^** | ***p* value^C^** |
| --- | --- | --- | --- | --- | --- |
| wild‑type | 95.1 ± 2.2 | 68.1 ± 6.0 | 628 |  |  |
| N2;*Ex1518[Pnhr-38::yc2.12]* | 92.0 ± 4.4 | 52.0 ± 4.6 | 200 | >0.05 | <0.001 |
| N2;*byEx1027[Pmec-3::yc2.12;unc-122::rfp]* | 92.2 ± 5.0 | 49.0 ± 7.9 | 266 | >0.05 | <0.001 |
| N2;*byEx1058[Pida-1::yc2.12;unc-122::rfp]* | 95.0 ± 3.2 | 50.4 ± 2.9 | 112 | >0.05 | <0.001 |
| *lin-15B(n765);bzIs18[Pmec-4::yc2.12+lin-15(+)]* | 94.0 ± 4.6 | 56.7 ± 8.8 | 226 | >0.05 | <0.01 |

Values reported are mean % ± SD %

n^A^ denotes number of animals tested, 3-17 independent assays were performed.

*p*^B^ values are compared to wild-type animals for the Tav response in the head.

*p*^C^ values are compared to wild-type animals for the Tav response in the tail.
