# Supplementary material for: Temperature- and Touch-Sensitive Neurons Couple CNG and TRPV Channel Activities to Control Heat Avoidance in Caenorhabditis elegans
Source: PLoS One. 2012 Mar 20;7(3):e32360. doi: 10.1371/journal.pone.0032360 (PMC3308950; doi:10.1371/journal.pone.0032360)
Supplement: Table S5 — OCR-2 and OSM-9 are required in the FLP and PHC neurons of C. elegans for the Tav response. Values reported are mean % ± SD %; nA denotes number of animals tested, 3–17 independent assays were performed; p B values are compared to wild-type animals for the Tav response in the head; p C values are compared to wild-type animals for the Tav response in the tail; p D values are compared to ocr-2(vs29)osm-9(ky10) for the Tav response in the head; p E values are compared to ocr-2(vs29)osm-9(ky10) for the Tav response in the tail. (DOCX) [file pone.0032360.s008.docx]

**Table S5. OCR-2 and OSM-9 are required in the FLP and PHC neurons of *C. elegans* for the Tav response**

| **Genotype** | **Tav response in the head** | **Tav response in the tail** | **n ^A^** | ***p* value^B^** | ***p* value^C^** |
| --- | --- | --- | --- | --- | --- |
| wild‑type | 95.1 ± 2.2 | 68.1 ± 6.0 | 628 |  |  |
| *osm-9(y26)* | 79.8 ± 6.7 | 42.8 ± 7.4 | 205 | <0.01 | <0.001 |
| *osm-9(ky10)* | 81.8 ± 9.1 | 41.3 ± 11.7 | 389 | <0.01 | <0.001 |
| *ocr-2(vs29)* | 86.5 ± 7.7 | 42.6 ± 18.7 | 276 | <0.01 | <0.001 |
| *ocr-2(yz5)* | 83.0 ± 9.9 | 41.8 ± 11.2 | 210 | <0.01 | <0.001 |
| *ocr-2(ak47)**osm-9(ky10)* | 78.6 ± 6.7 | 20.5 ± 2.5 | 254 | <0.01 | <0.001 |
| *ocr-2(vs29)osm-9(ky10)* | 73.5 ± 6.1 | 14.5 ± 7.7 | 201 | <0.01 | <0.001 |
| *ocr‑2osm‑9;byEx772[Pocr‑2::ocr‑2;Posm‑9::osm‑9;myo‑2::mCherry]* | 96.9 ± 3.8 | 51.8 ± 6.8 | 86 | <0.001^D^ | <0.001^E^ |
| *ocr-2osm-9;byEx773[Pocr-2::ocr-2;Posm-9::osm-9;myo‑2::mCherry]* | 96.3 ± 5.0 | 59.8 ± 15.5 | 81 | <0.001^D^ | <0.001^E^ |
| *ocr-2osm-9;byEx1086[Posm-9::osm-9::gfp;unc-122::rfp]* | 87.7 ± 10.9 | 33.7 ± 5.4 | 225 | <0.01^D^ | <0.05^E^ |
| *ocr-2osm-9;byEx1088[Pocr-2::ocr-2::gfp;unc-122::rfp]* | 93.3 ± 6.3 | 41.3 ± 7.0 | 194 | <0.001^D^ | <0.01^E^ |
| *ocr‑2osm‑9;byEx1022[Pmec‑3::ocr‑2;Pmec‑3::osm‑9;unc‑122::rfp]* | 89.7 ± 4.6 | 37.7 ± 7.5 | 192 | <0.001^D^ | <0.01^E^ |
| *ocr‑2osm‑9;byEx1023[Pmec‑3::ocr‑2;Pmec‑3::osm‑9;unc‑122::rfp]* | 88.8 ± 1.5 | 36.2 ± 7.4 | 140 | <0.001^D^ | <0.01^E^ |
| *ocr-2osm-9;byEx1084[Pmec-3::ocr-2::gfp;unc-122::rfp]* | 91.1 ± 2.0 | 30.3 ± 7.7 | 210 | <0.001^D^ | >0.05^E^ |
| *ocr-2osm-9;byEx1085[Pmec-3::ocr-2::gfp;unc-122::rfp]* | 86.2 ± 4.5 | 18.8 ± 3.4 | 188 | <0.01^D^ | >0.05^E^ |
| *ocr-2osm-9;byEx1087[Pmec-3::osm-9::gfp;unc-122::rfp]* | 91.7 ± 4.0 | 24.9 ± 6.6 | 188 | <0.001^D^ | >0.05^E^ |
| *ocr-4(vs137)* | 91.9 ± 4.9 | 64.1 ± 6.7 | 319 | >0.05 | >0.05 |
| *trp-1(ok323)* | 94.8 ± 2.7 | 65.1 ± 5.5 | 113 | >0.05 | >0.05 |
| *trp-2(gk298)* | 98.5 ± 1.0 | 74.4 ± 7.1 | 198 | >0.05 | >0.05 |
| *trp-4(gk341)* | 95.5 ± 2.4 | 70.1 ± 9.2 | 75 | >0.05 | >0.05 |
| *trpa-1(ok999)* | 96.2 ± 0.8 | 61.4 ± 1.2 | 107 | >0.05 | >0.05 |

Values reported are mean % ± SD %

n^A^ denotes number of animals tested, 3-17 independent assays were performed.

*p*^B^ values are compared to wild-type animals for the Tav response in the head.

*p*^C^ values are compared to wild-type animals for the Tav response in the tail.

*p*^D^ values are compared to *ocr-2(vs29)osm-9(ky10)* for the Tav response in the head.

*p*^E^ values are compared to *ocr-2(vs29)osm-9(ky10)* for the Tav response in the tail.
