# Supplementary material for: Temperature- and Touch-Sensitive Neurons Couple CNG and TRPV Channel Activities to Control Heat Avoidance in Caenorhabditis elegans
Source: PLoS One. 2012 Mar 20;7(3):e32360. doi: 10.1371/journal.pone.0032360 (PMC3308950; doi:10.1371/journal.pone.0032360)
Supplement: Table S6 — TAX-2 and TAX-4 are required in the AFD neurons in the Tav response in the anterior part of C. elegans . Values reported are mean % ± SD %; nA denotes number of animals tested, 3–17 independent assays were performed; ND: not determined; p B values are compared to wild-type animals for the Tav response in the head; p C values are compared to wild-type animals for the Tav response in the tail; p D values are compared to tax-4(p678);byEx925[myo-2::mCherry]; p E values are compared to tax-2(p671);byEx925[myo-2::mCherry]. (DOCX) [file pone.0032360.s009.docx]

**Table S6. TAX-2 and TAX-4 are required in the AFD neurons in the Tav response in the anterior part of *C. elegans***

| **Genotype** | **Tav response in the head** | **Tav response in the tail** | **n^A^** | ***p* value^B^** | ***p* value^C^** |
| --- | --- | --- | --- | --- | --- |
| wild‑type | 95.2 ± 2.0 | 68.1 ± 6.0 | 628 |  |  |
| *tax-4(p678)* | 70.7 ± 8.1 | 42.3 ± 1.7 | 187 | <0.001 | <0.001 |
| *tax-4(ks28)* | 73.4 ± 3.0 | 61.2 ± 3.6 | 162 | <0.001 | >0.05 |
| *tax-4(ks11)* | 64.6 ± 4.5 | 49.2 ± 3.4 | 154 | <0.001 | <0.001 |
| *tax-2(p671)* | 64.5 ± 7.0 | 43.5 ± 9.7 | 235 | <0.001 | <0.001 |
| *tax-2(ks10)* | 70.6 ± 5.6 | ND | 136 | <0.001 |  |
| *tax-2(ks31)* | 66.0 ± 2.6 | ND | 154 | <0.001 |  |
| *cng-1(jh111)* | 97.4 ± 0.4 | ND | 78 | >0.05 |  |
| *cng-3(jh113)* | 92.8 ± 4.4 | ND | 139 | >0.05 |  |
| *tax-2(p671);tax-4(p678)* | 42.8 ± 16.3 | 36.0 ± 7.6 | 280 | <0.001 | <0.001 |
| BR5256;*byEx851[Pgcy-8::DTA;myo-2::mCherry]* | 42.3 ± 16.3 | 61.2 ± 3.2 | 83 | <0.001 | >0.05 |
| *tax-2(p671);tax-4(p678)*; BR5256;*byEx851* | 42.4 ± 7.4 | 37.1 ± 10.8 | 217 | <0.001 | <0.001 |
| *ocr-2(vs29)osm-9(ky10)* | 73.5 ± 6.1 | 14.5 ± 7.7 | 201 | <0.01 | <0.001 |
| *tax-2;tax-4; ocr-2osm-9* | 41.7 ± 4.8 | 15.6 ± 6.9 | 371 | <0.001 | <0.001 |
| *cng-3(jh113);cng-1(jh111)* | 93.6 ± 2.1 | ND | 130 | >0.05 |  |
| *tax-4(p678);byEx925[myo-2::mCherry]* | 51.6 ± 13.5 | 46.2 ± 4.8 | 143 | <0.001 | <0.001 |
| *tax-4(p678);byEx836[Podr-4::tax-4;,myo-2::mCherry]* | 53.6 ± 10 | 46.8 ± 11 | 185 | >0.05^D^ | >0.05^D^ |
| *tax-4(p678);byEx774[Ptax-4::tax-4;,myo-2::mCherry]* | 86.0 ± 1.6 | 62.2 ± 3.0 | 100 | <0.001^D^ | <0.001^D^ |
| *tax-4(p678);byEx876[Ptax-4::tax-4;myo-2::mCherry]* | 88.6 ± 6.4 | 61.5 ± 2.1 | 67 | <0.001^D^ | <0.001^D^ |
| *tax-4(p678);byEx776[Pgcy-8::tax-4;myo-2::mCherry]* | 85.8 ± 8.6 | 39.9 ± 8.8 | 50 | <0.001^D^ | >0.05^D^ |
| *tax-4(p678);byEx878[Pgcy-8::tax-4;myo-2::mCherry]* | 82.1 ± 6.2 | 35.4 ± 2.9 | 52 | <0.001^D^ | >0.05^D^ |
| *tax-2(p671);byEx925[myo-2::mCherry]* | 44.4 ± 4.6 | 50.6 ± 2.4 | 154 | <0.001 | <0.001 |
| *tax-2(p671);byEx926[Podr-4::tax-2;myo-2::mCherry]* | 45.4 ± 9.5 | 48.8 ± 8.2 | 147 | >0.05^E^ | >0.05^E^ |
| *tax-2(p671);byEx834[Ptax-2::tax-2;myo-2::mCherry]* | 69.5 ± 3.8 | 60.3 ± 4.8 | 136 | <0.001^E^ | >0.05^E^ |
| *tax-2(p671);byEx835[Ptax-2::tax-2;myo-2::mCherry]* | 75.2 ± 8.3 | 54.4 ± 7.3 | 68 | <0.001^E^ | >0.05^E^ |
| *tax-2(p671);byEx808[Pgcy-8::tax-2;myo-2::mCherry]* | 76.2 ± 9.7 | 35.1 ± 8.5 | 161 | <0.001^E^ | >0.05^E^ |
| *tax-2(p671);byEx809[Pgcy-8::tax-2;myo-2::mCherry]* | 76.3 ± 8.1 | 33.2 ± 3.2 | 137 | <0.001^E^ | >0.05^E^ |
| *gcy-12(nj10)* | 68.5 ± 2.9 | 61.0 ± 4.3 | 193 | <0.001 | >0.05 |
| *gcy-8(oy44)* | 90.7 ± 8.8 | ND | 101 | >0.05 |  |
| *gcy-18(nj38)* | 94.0 ± 2.6 | ND | 98 | >0.05 |  |
| *gcy-23(nj37)* | 92.7 ± 5.0 | ND | 112 | >0.05 |  |
| *gcy-8;gcy-18;gcy-23* | 76.0 ± 6.6 | ND | 116 | <0.01 |  |
| *srtx-1(tm2064)* | 96.6 ± 2.9 | ND | 137 | >0.05 |  |

Values reported are mean % ± SD %

ND: not determined

n^A^ denotes number of animals tested, 3-17 independent assays were performed.

*p*^B^ values are compared to wild-type for the Tav response in the head.

*p*^C^ values are compared to wild-type for the Tav response in the tail.

*p*^D^ values are compared to *tax-4(p678);byEx925[myo-2::mCherry]*.

*p*^E^ values are compared to *tax-2(p671);byEx925[myo-2::mCherry]*.
