# Supplementary material for: Temperature- and Touch-Sensitive Neurons Couple CNG and TRPV Channel Activities to Control Heat Avoidance in Caenorhabditis elegans
Source: PLoS One. 2012 Mar 20;7(3):e32360. doi: 10.1371/journal.pone.0032360 (PMC3308950; doi:10.1371/journal.pone.0032360)
Supplement: Table S7 — Strains used for laser-ablation to label neurons. (DOCX) [file pone.0032360.s010.docx]

Table S7. Strains used for laser‑ablation to label neurons

| **Strains** | **Genotype** | **neurons** |
| --- | --- | --- |
| PY1322 | *oyIs18[Pgcy-8::**gfp]* | AFD |
| TU2562 | *dpy-20(e1282);uIs22[Pmec-3::gfp+ dpy-20(+)]* | FLP,ALM,AVM,PVM,PLM |
| BR5256 | *dpy-20(e1282); uIs22[Pmec-3::gfp+ dpy-20(+)];oyIs18[Pgcy-8:: gfp]* | AFD,FLP,ALM,AVM,PVM,PLM |
| OH1098 | *otIs133[Pttx-3::rfp+unc-4(+)]* | AIY |
| ZW281 | *lin-15B(n765);zwEx101[Pinx-1::gfp+lin-15(+)]* | AIB |
| CX3465 | *kyIs39[Psra-6:: gfp +lin-15(+)]* | ASH |
| CX3260 | *kyIs37[Podr-10:: gfp,lin-15]* | AWA |
| OH7253 | *zdIs13[Ptph-1:: gfp];otEx3165[Punc-120::hif-1(p621A)+ttx-3::rfp]* | ADF |
| BR5393 (OH4770) | *otIs24 [Psre-1:: gfp]* | ADL |
| CX3553 | *lin-15B(n765)kyIs104[Pstr-1:: gfp]* | AWB |
| NY2064 | *ynIs64[Pflp-17:: gfp];him-5(e1490)* | BAG |
| BL5752 | *inIs181;inIs182[ida-1:: gfp]* | PHC |
| CX3716 | *lin-15B(n765)kyIs141[osm-9:: gfp5+lin-15(+)]* | PHA,PHB |
| OH1422 | *otIs138[ser-2prom3::gfp+rol-6]* | PVD |
| VM484 | *akIs3[Pnmr-1:: gfp]* | PVC |
| VM141 | *akEx32[Pglr-4:: gfp]* | DVA |
