# Supplementary material for: Temperature- and Touch-Sensitive Neurons Couple CNG and TRPV Channel Activities to Control Heat Avoidance in Caenorhabditis elegans
Source: PLoS One. 2012 Mar 20;7(3):e32360. doi: 10.1371/journal.pone.0032360 (PMC3308950; doi:10.1371/journal.pone.0032360)
Supplement: Table S8 — Plasmids constructed in this study. (DOCX) [file pone.0032360.s011.docx]

Table S8. Plasmids constructed in this study

| **Plasmid** | **Description** | **Insert and Vector** |
| --- | --- | --- |
| **pBY2974** | *Pgcy-8::tax-4::GFP* | *tax-4* cDNA cloned into pBY566 with KpnI/XmaI |
| **pBY3114** | *Podr-4::tax-4::GFP* | *odr-4* promoter sequence 4.6kb upstream the coding region cloned into pBY2974 with SphI/XmaI |
| **pBY3117** | *Pgcy-8::tax-2::GFP* | *tax-2* cDNA cloned into pBY566 with KpnI/XmaI |
| **pBY3115** | *Podr-4::tax-2::GFP* | *odr-4* promoter sequence 4.6kb upstream the coding region cloned into pBY3117 with SphI/XmaI |
| **pBY3118** | *Pgcy-8::DT‑A(WT)* | DT-A(WT) cloned into pBY566 with KpnI/XmaI |
| **pBY3125** | *Podr-4::GFP* | *Podr-4* promoter sequence cloned into pBY566 with SphI/XmaI |
| **pBY3269** | *Podr-3::DT‑A(WT)* | *Podr-3* promoter sequence cloned into pBY3118 with SphI/XmaI |
| **pBY3270** | *Pmec-3::YC2.12* | *Pmec-3* promoter 2kb upstream and the coding region to 4aa of the 5 exon cloned into PKDK189 with XmaI/NheI |
| **pBY3276** | *Pgcy-8::YC4.12* | *Pgcy-8* promoter sequence cloned into PKDK153 with SbfI/Acc65I |
| **pBY3278** | *Pida-1::YC2.12* | YC2.12 cut out of PKDK189 cloned into pEM-1 with SbfI/SpeI, then *Pida-1* cloned with FseI/AscI, cut LoxPStopLoxP out with NheI |
| **pBY3279** | *Pmec-3::ocr-2::GFP* | *ocr-2* cDNA cloned into pEGFP-N1 with XhoI/SmaI, *Pmec-3* cloned upstream of it with Eco47II/XhoI |
| **pBY3280** | *Pmec-3::osm-9::GFP* | *osm-9* cDNA cloned into pEGFP-N1 with XhoI/SacII, *Pmec-3* cloned upstream of it with Eco47II/XhoI |
