# Supplementary material for: Temperature- and Touch-Sensitive Neurons Couple CNG and TRPV Channel Activities to Control Heat Avoidance in Caenorhabditis elegans
Source: PLoS One. 2012 Mar 20;7(3):e32360. doi: 10.1371/journal.pone.0032360 (PMC3308950; doi:10.1371/journal.pone.0032360)
Supplement: Table S9 — Transgenic strains used in this study. (DOCX) [file pone.0032360.s012.docx]

Table S9. Transgenic strains used in this study

| **Strains** | **Genotype** |
| --- | --- |
| BR5187 | *ocr-2(vs29)osm-9(ky10);byEx772[Pocr-2::ocr-2::gfp;Posm-9::osm-9::gfp;myo-2::mCherry]* |
| BR5195 | *ocr-2(vs29)osm-9(ky10);byEx773[Pocr-2::ocr-2::gfp;Posm-9::osm-9::gfp;myo-2::mCherry]* |
| BR5848 | *ocr-2(vs29)osm-9(ky10);byEx1022[Pmec-3::ocr-2::gfp;Pmec-3::osm-9::gfp;unc-122::rfp]* |
| BR5849 | *ocr-2(vs29)osm-9(ky10);byEx1023[Pmec-3::ocr-2::gfp;Pmec-3::osm-9::gfp;unc-122::rfp]* |
| BR5723 | *tax-4(p678);byEx925[myo-2::mCherry]* |
| BR5602 | *tax-4(p678);byEx836[Podr-4::tax-4::gfp;myo-2::mCherry]* |
| BR4875 | *tax-4(p678);byEx774[Ptax-4::tax-4::gfp;myo-2::mCherry]* |
| BR4876 | *tax-4(p678);byEx876[Ptax-4::tax-4::gfp;myo-2::mCherry]* |
| BR5394 | *tax-4(p678);byEx776[Pgcy-8::tax-4::gfp;myo-2::mCherry]* |
| BR5395 | *tax-4(p678);byEx878[Pgcy-8::tax-4::gfp;myo-2::mCherry]* |
| BR5722 | *tax-2(p671);byEx925[myo-2::mCherry]* |
| BR5725 | *tax-2(p671);byEx926[Podr-4::tax-2::gfp;myo-2::mCherry]* |
| BR5600 | *tax-2(p671);byEx834[Ptax-2::tax-2::gfp;myo-2::mCherry]* |
| BR5601 | *tax-2(p671);byEx835[Ptax-2::tax-2::gfp;myo-2::mCherry]* |
| BR5549 | *tax-2(p671);byEx808[Pgcy-8::tax-2::gfp;myo-2::mCherry]* |
| BR5550 | *tax-2(p671);byEx809[Pgcy-8::tax-2::gfp;myo-2::mCherry]* |
| BR5634 | BR5256*;byEx851[Pgcy-8::DTA;myo-2::mCherry]* |
| BR5635 | BR5256*;byEx852[Pgcy-8::DTA;myo-2::mCherry]* |
| BR5410 | N2;*byEx925[myo-2::mCherry]* |
| BR5852 | N2;*byEx1025[Podr-3::DTA;Podr-4::gfp;myo-2::mCherry]* |
| BR5853 | N2;*byEx1026[Podr-3::DTA;Podr-4::gfp;myo-2::mCherry]* |
| IK699 | N2;*Ex1518[Pnhr-38::yc2.12]* |
| BR5854 | N2;*byEx1027[Pmec-3::yc2.12;unc-122::rfp]* |
| BR5916 | N2;*byEx1058[Pida-1::yc2.12;unc-122::rfp]* |
| ZB1057 | lin-15B(n765);bzIs18[Pmec-4::yc2.12+lin-15(+)] |
