# Supplementary material for: Temperature- and Touch-Sensitive Neurons Couple CNG and TRPV Channel Activities to Control Heat Avoidance in Caenorhabditis elegans
Source: PLoS One. 2012 Mar 20;7(3):e32360. doi: 10.1371/journal.pone.0032360 (PMC3308950; doi:10.1371/journal.pone.0032360)
Supplement: Table S10 — References for mutant strains. (DOCX) [file pone.0032360.s013.docx]

Table S10. References for mutant strains

| **Strains** | **Genotype** | **Reference** |
| --- | --- | --- |
| PR767 | *ttx-1(p767)* | Satterlee, J.S.*, et al.*^9^ |
| MT1859 | *unc-86(n846)* | Baumeister, R., Liu, Y. & Ruvkun, G.^17^ |
| CB1338 | *mec-3(e1338)* | Xue, D., Finney, M., Ruvkun, G. & Chalfie, M.^10^ |
| MT2247 | *egl-44(n1080)* | Wu, J., Duggan, A. & Chalfie, M.^11^ |
| MT2316(BR5551) | *egl-46(n1127)* | Wu, J., Duggan, A. & Chalfie, M.^11^ |
| MT3179 | *sem-4(n1378)* | Basson, M. & Horvitz, H.R.^16^ |
| TU38 | *deg-1(u38)* | Chalfie, M. & Wolinsky, E.^19^ |
| OH161 | *ttx-3(ot22)* | Hobert, O.*, et al.*^36^ |
| OH8 | *ttx-3(mg158)* | Hobert, O.*, et al.*^36^ |
| JY190 | *osm-9(yz6)* | Zhang, S., Sokolchik, I., Blanco, G. & Sze, J.Y.^43^ |
| CX2327 | *osm-9(ky10)* | Colbert, H.A., Smith, T.L. & Bargmann, C.I.^37^ |
| LX671 | *ocr-2(vs29)* | Jose, A.M., Bany, I.A., Chase, D.L. & Koelle, M.R.^26^ |
| JY243 | *ocr-2(yz5)* | Zhang, S., Sokolchik, I., Blanco, G. & Sze, J.Y.^43^ |
| CX4544 | *ocr-2(ak47)* | Tobin, D.*, et al.*^25^ |
| BR4008 | *ocr-2(ak47)osm-9(ky10)* | this paper |
| LX842 | *ocr-2(vs29)osm-9(ky10)* | Jose, A.M., Bany, I.A., Chase, D.L. & Koelle, M.R.^26^ |
| CX4534 | *ocr-1(ak46)* | Tobin, D.*, et al.*^25^ |
| RB1374(BR4176) | *ocr-3(ok1559)* | this paper |
| LX950 | *ocr-4(vs137)* | Jose, A.M., Bany, I.A., Chase, D.L. & Koelle, M.R.^26^ |
| VC160(BR4024) | *trp-1(ok323)* | this paper |
| VC602 | *trp-2(gk298)* | this paper |
| VC818(BR4025) | *trp-4(gk341)* | this paper |
| RB1052 | *trpa-1(ok999)* | this paper |
| PR678(BR5083) | *tax-4(p678)* | Coburn, C.M., Mori, I., Ohshima, Y. & Bargmann, C.I.^38^ |
| FK103 | *tax-4(ks28)* | Komatsu, H., Mori, I., Rhee, J.S., Akaike, N. & Ohshima, Y.^40^ |
| FK129 | *tax-4(ks11)* | Komatsu, H., Mori, I., Rhee, J.S., Akaike, N. & Ohshima, Y.^40^ |
| PR671(BR5459) | *tax-2(p671)* | Coburn, C.M. & Bargmann, C.I. A^28^ |
| FK100 | *tax-2(ks10)* | Coburn, C.M. & Bargmann, C.I. A^28^ |
| FK104 | *tax-2(ks31)* | Coburn, C.M. & Bargmann, C.I. A^28^ |
| KJ461 | *cng-1(jh111)* | Cho, S.W., Cho, J.H., Song, H.O. & Park, C.S.^29^ |
| KJ462 | *cng-3(jh113)* | Cho, S.W., Choi, K.Y. & Park, C.S.^30^ |
| BR5514 | *tax-2(p671);tax-4(p678)* | this paper |
| BR6044 | *tax-2(p671);tax-4(p678);ocr-2(vs29)osm-9(ky10)* | this paper |
| KJ5560 | *cng-3(jh113);cng-1(jh111)* | Cho, S.W., Cho, J.H., Song, H.O. & Park, C.S.^29^ |
| IK800 | *gcy-8(oy44)* | Inada, H.*, et al.*^12^ |
| IK429 | *gcy-18(nj38)* | Inada, H.*, et al.*^12^ |
| IK427 | *gcy-23(nj37)* | Inada, H.*, et al.*^12^ |
| IK597 | *gcy-8(oy44);gcy-18(nj38);gcy-23(nj37)* | Inada, H.*, et al.*^12^ |
| IK212 | *gcy-12(nj10)* | Inada, H.*, et al.*^12^ |
| CB450 | *unc-13(e450)* | Madison, J.M., Nurrish, S. & Kaplan, J.M.^22^ |
| DA509 | *unc-31(e928)* | Speese, S.*, et al.*^23^ |
